# Tetryl‐Tetrylene Addition to Phenylacetylene

**DOI:** 10.1002/chem.202005119

**Published:** 2021-02-04

**Authors:** Jakob‐Jonathan Maudrich, Fatima Diab, Sebastian Weiß, Magda Zweigart, Klaus Eichele, Hartmut Schubert, Robert Müller, Martin Kaupp, Lars Wesemann

**Affiliations:** ^1^ Institut für Anorganische Chemie Universität Tübingen Auf der Morgenstelle 18 72076 Tübingen Germany; ^2^ Institut für Chemie Theoretische Chemie/Quantenchemie Sekr. C7 Technische Universität Berlin Straße des 17. Juni 135 10623 Berlin Germany

**Keywords:** germaniumhydrides, NMR chemical shift calculations, stannyl cations, tetrylenes, tinhydrides

## Abstract

Phenylacetylene adds [Ar*GeH_2_‐SnAr’], [Ar*GeH_2_‐PbAr’] and [Ar'SnH_2_‐PbAr*] at rt in a regioselective and stereoselective reaction. The highest reactivity was found for the stannylene, which reacts immediately upon addition of one equivalent of alkyne. However, the plumbylenes exhibit addition to the alkyne only in reaction with an excess of phenylacetylene. The product of the germylplumbylene addition reacts with a second equivalent of alkyne and the product of a CH‐activation, a dimeric lead acetylide, were isolated. In the case of the stannylplumbylene the *trans*‐addition product was characterized as the kinetically controlled product which isomerizes at rt to yield the *cis*‐addition product, which is stabilized by an intramolecular Sn–H–Pb interaction. NMR chemical shifts of the olefins were investigated using two‐ and four‐component relativistic DFT calculations, as spin–orbit effects can be large. Hydride abstraction was carried out by treating [Ar'SnPhC=CHGeH_2_Ar*] with the trityl salt [Ph_3_C][Al(OC{CF_3_})_4_] to yield a four membered ring cation.

## Graphical abstract

**Phenylacetylene adds** [Ar*GeH_2_‐SnAr’], [Ar*GeH_2_‐PbAr’] and [Ar'SnH_2_‐PbAr*] in a regioselective and stereoselective reaction. NMR chemical shifts of the olefins were investigated using two‐ and four‐component relativistic DFT calculations, as spin–orbit effects can be large. Hydride abstraction was carried out by treating [Ar'SnPhC=CHGeH_2_Ar*] with the trityl salt [Ph_3_C][Al(OC{CF_3_}_3_)_4_] to yield cationic germastannacyclobutene.

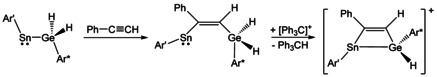

## Introduction

**Scheme 1:**
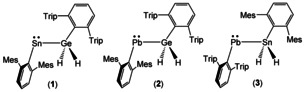
Tetryl‐tetrylenes.

The element–element addition to alkynes is an attractive synthetic method for the synthesis of substituted olefins.^[1]^ This regioselective as well as stereoselective addition to alkynes results in the case of heteroelement bond addition E−E’ (E−E’: Si−Ge, Ge−Sn) in formation of *syn*‐addition products with two different substituents.^[2]^ Various examples for this reaction have been presented in the literature and are topics of review articles.[^1a^, ^1b^, ^1c^] In many cases this reaction is catalyzed by transition metals like Ni, Pd and Pt. Transition metal catalyzed addition reactions were also found to form mixtures between *E*‐ and *Z*‐isomers as products of alkyne addition. Marschner et al. studied the synthesis of vinyl germylenes reacting silyl substituted germylenes with phenylalkyne.^[2o]^ Aldridge et al. presented the regioselective *syn*‐addition of boryl‐stannylene as well as boryl‐germylene to substituted alkynes.^[3]^ Power et al. investigated the reaction of a low valent organotin hydride with phenylalkyne and characterized a *syn*‐addition of a stannylstannylene at the alkyne moiety.^[2i]^ These hydrides are very reactive compounds, which exhibit hydroelementation reactions, activation of small molecules, and were shown to act as catalysts in organic transformation reactions.[^2i^, ^4^] We are interested in the hydride chemistry of the heavy Group 14 elements and have reported recently the deprotonation of organoelement trihydrides of germanium and tin.^[5]^ The anionic deprotonation products serve as versatile starting materials for nucleophilic substitution reactions. Thus as the first examples of nucleophilic substitution with these anionic organoelement dihydrides [ArEH_2_]^−^ (E=Ge, Sn; Ar=Ar*, Ar’) in reaction with electrophiles of low valent Group 14 elements we published formation of tetryl‐tertrylenes: Ar*GeH_2_‐SnAr’ (**1**), Ar*GeH_2_‐PbAr’ (**2**), Ar'SnH_2_‐PbAr* (**3**); Ar*=2,6‐Trip_2_C_6_H_3_, Trip=2,4,6‐triisopropylphenyl; Ar’=2,6‐Mes_2_C_6_H_3_, Mes=2,4,6‐trimethylphenyl (Scheme 1). Here we would like to present the reactions of the reported tetryl‐tetrylenes with phenylalkyne.

## Results and Discussion

**Scheme 2:**
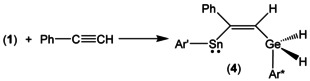
Addition of germylstannylene **1** to phenylalkyne.

Germylstannylene **1**, germylplumbylene **2** and stannylplumbylene **3** were treated with phenylacetylene. Reaction of **1** with equimolar amounts of phenylalkyne is fast at rt, the color of the mixture changes from violet to pink and quantitative formation of product **4** was observed by ^1^H NMR spectroscopy (Scheme 2). In the ^1^H NMR spectrum the signal for the GeH_2_ unit was found at 3.76 ppm and can be compared with signals found for hydrides presented in the literature: Ar*GeH_3_ 3.61 ppm, Ar^#^_2_GeH_2_ 4.61 ppm (Ar^#^=C_6_H_3_‐2,6‐(C_6_H_2_‐2,4,6‐Me_3_)_2_),^[6]^ (*o‐t*BuC_6_H_4_)_3_GeH 5.95 ppm.^[7]^ The olefinic proton Ge−CH=C exhibits a signal at 6.79 ppm, comparable to signals found for olefinic protons in triorganogermyl substituted olefins.^[8]^ The ^119^Sn NMR signal of the arylvinylstannylene **4** with 1630 ppm is characteristic of a monomeric stannylene and can be compared with the signal found for the diphenylacetylene insertion product of [Ar*SnH]_2_ (1573.9 ppm).^[2i]^

**Figure 1:**
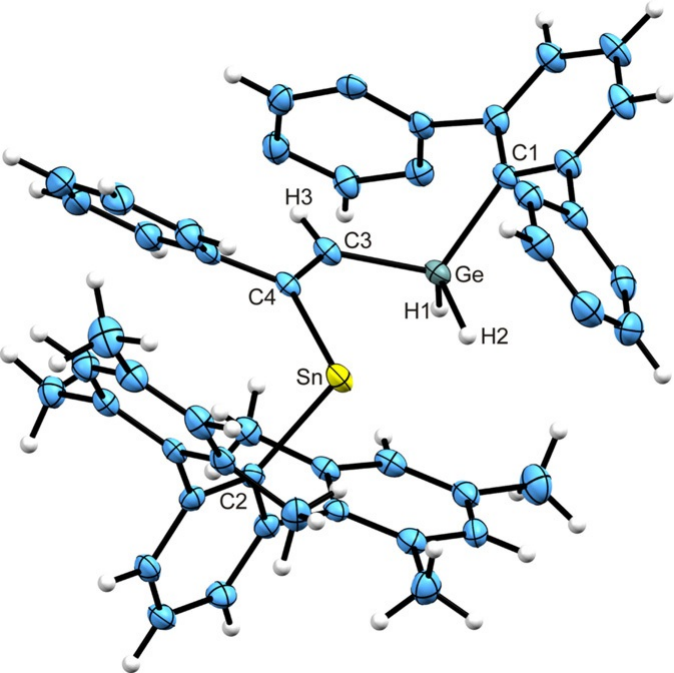
ORTEP of **4**. Hydrogen atoms were placed in idealized positions. *i*Pr groups are omitted for clarity. Ellipsoids at 50 % probability. Interatomic distances [Å] and angles [deg]: Sn‐C2 2.213(4), Sn‐C4 2.214(4), Ge‐C3 1.931(4), Ge‐C1 1.973(4), C3‐C4 1.344(5), Ge‐H1 1.48(5), Ge‐H2 1.45(5), C2‐Sn‐C4 99.34(13), C3‐Ge‐C1 113.80(16), C3‐C4‐Sn 124.0(3), C4‐C3‐Ge 126.1(3).

Crystals suitable for single crystal structure analysis were obtained from a hexane solution of **4** at −40 °C. In Figure 1 an ORTEP of the structure of **4** in the solid state is shown. Selected interatomic distances and angles are listed. Details of the crystal structure analysis are placed in the Supporting Information. Vinylstannylenes were already presented in the literature and exhibit Sn−C(vinyl) distances of 2.2020(17) and 2.198(3) Å.^[2i]^ The Sn−C(vinyl) distance in **4** of 2.214(4) Å is only slightly longer. The Ge−C(vinyl) bond length of 1.931(4) Å can be compared with distances reported for Ph_3_Ge(CH=CH_2_) [Ge−C 1.942(2) Å], Ph_3_Ge(CH=CHPh) [Ge−C 1.933(3) Å] and *cis*‐(Me_3_Ge)_2_(Ph_2_C_2_) [Ge−C 1.977(3) Å].[^8^, ^9^]

**Scheme 3:**
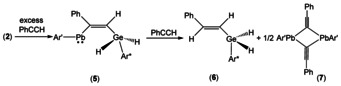
Reaction of germylplumbylene **2** with an excess of phenylalkyne.

Germylplumbylene **2** does not react at rt with equimolar amounts of phenylacetylene over a period of several days. Therefore **2** was treated with an excess of alkyne at rt and in the ^1^H NMR spectrum formation of the addition product **5** was observed. However, the addition product could not be isolated. Instead, the products of a CH‐activation reaction, **6** and **7**, with a further equivalent of alkyne were characterized (Scheme 3). Diorganogermanium dihydride **6** was characterized by NMR spectroscopy, and dimeric plumbylene **7** was characterized by single crystal structure analysis (Figure 3). To isolate addition product **5** the reaction mixture with the excess of phenylalkyne was cooled to −40 °C after 5 h stirring at rt. A small amount of violet crystals of **5** was obtained after crystallization from hexane. Because we isolated the temperature sensitive addition product **5** only in low yield, we were not able to obtain an elemental analysis.

**Figure 2:**
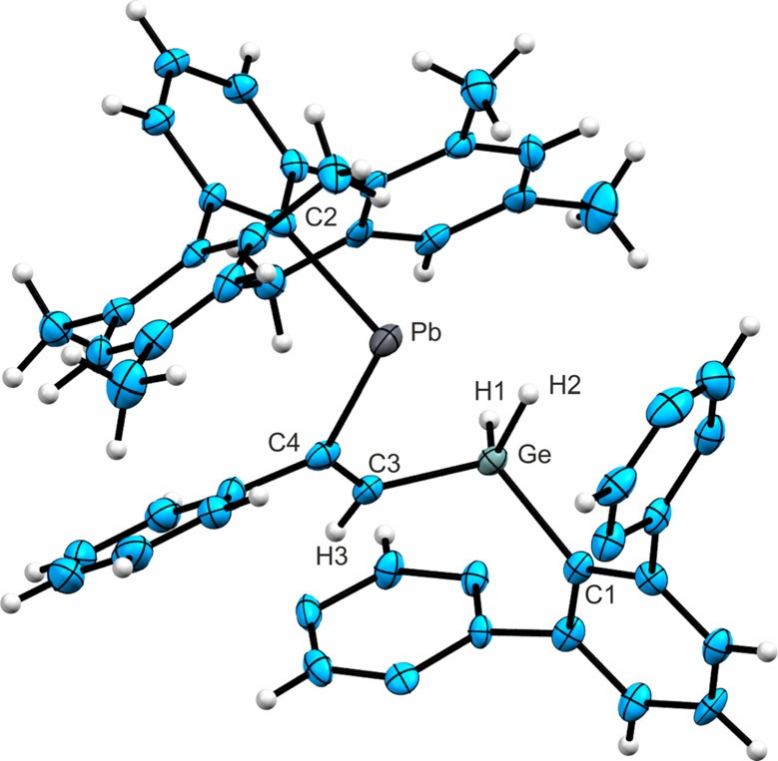
ORTEP of **5**. Hydrogen atoms were placed in idealized positions. *i*Pr groups are omitted for clarity. Ellipsoids at 50 % probability. Interatomic distances [Å] and angles [deg]: Pb‐C2 2.319(7), Pb‐C4 2.318(7), Ge‐C3 1.922(7), Ge‐C1 1.986(7), C3‐C4 1.352(10), Ge‐H1 1.41(3), Ge‐H2 1.41(3), C2‐Pb‐C4 98.4(2), C3‐Ge‐C1 115.1(3), C3‐C4‐Pb 122.3(6), C4‐C3‐Ge 126.5(6).

The result of the crystal structure analysis of **5** is shown in Figure 2. Details of the crystal structure analysis can be found in the Supporting Information. An arylvinylplumbylene was not found in the CCDC database. Cyclic molecules with lead carrying a vinyl group as well as phenyl substituents were found in the literature.^[10]^ In these compounds the Pb−C(vinyl) distances 2.329(3), 2.306(5), 2.320(5) Å, and the Pb−C(aryl) distance 2.360(3) Å are in a comparable range in comparison to molecule **5** Pb−C(vinyl) 2.318(7) Å, Pb−C(aryl) 2.319(7) Å.^[10]^

In solution ^1^H, ^13^C{^1^H} and ^207^Pb{^1^H} NMR spectra exhibit signals which are characteristic of a low valent lead compound. The signal which was found in the ^207^Pb NMR spectrum for plumbylene **5** (7591 ppm) is indicative of a monomeric plumbylene and lies in the range of signals found for plumbylenes **2** (9183 ppm) and **3** (8885 ppm) (see Scheme 1 for **2** and **3**).[^5f^, ^11^] Very remarkable is the ^1^H NMR chemical shift, 13.10 ppm, of the vinyl proton Ge−C*H*=CPh−Pb next to the Ge atom. The influence of relativistic effects of a heavy atom on the chemical shift of a neighboring light atom, in particular caused by spin–orbit coupling (the spin–orbit‐induced heavy‐atom on light‐atom shielding, SO‐HALA effect), received substantial interest over the past decades.^[12]^ In compound **5** the CH unit is not directly bonded to the lead atom and so this unusual shift is primarily due to a three bond SO‐HALA effect.^[13]^ Furthermore the signals of the carbon atoms connected to the lead atom were also found at high frequencies [263.1 ppm (s, Pb−*C*_Ph(Mes)2_), 273.6 ppm (s, *C*(Ph)−Pb)] (vide infra).^[14]^

**Figure 3:**
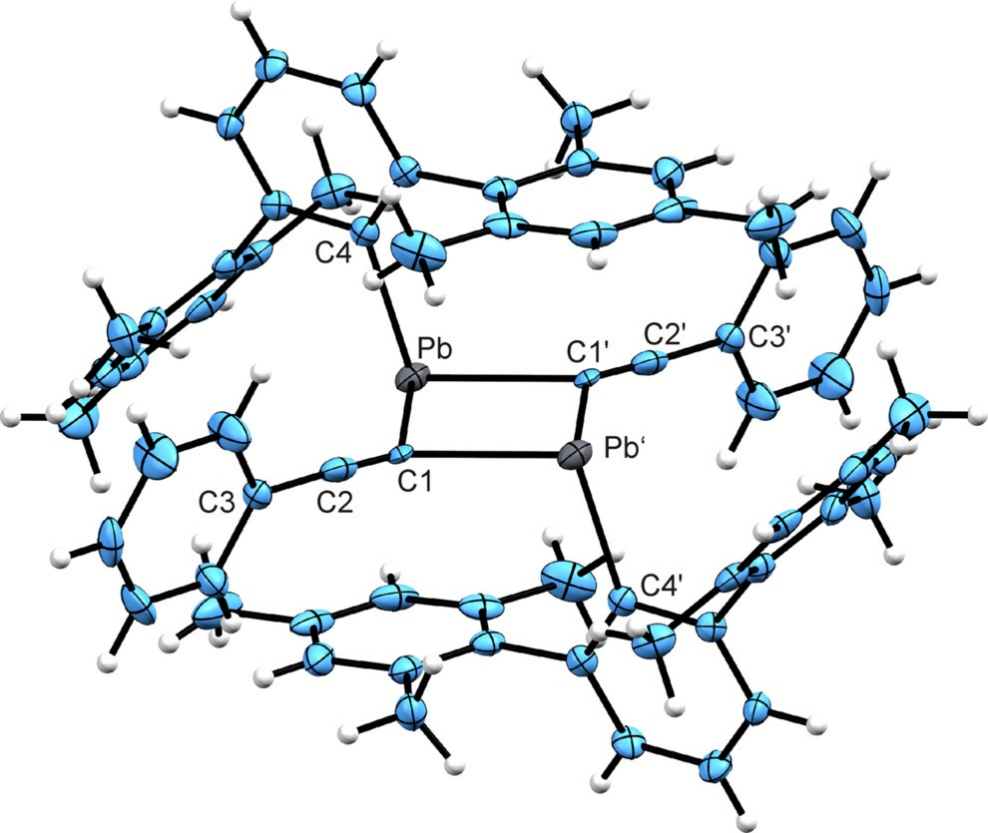
ORTEP of **7**. Hydrogen atoms were placed in idealized positions. Ellipsoids at 50 % probability. Interatomic distances [Å] and angles [deg]: Pb‐C1 2.467(5), Pb‐C1’ 2.498(5), C1‐C2 1.100(7), C2‐C3 1.491(7), Pb‐C4 2.343(5), Pb‐C1‐Pb’ 92.2(2), C1‐Pb‐C1’ 87.8(2), C4‐Pb‐C1 92.6(2), C4‐Pb‐C1’ 104.4(2), C2‐C1‐Pb 130.7(4), C2‐C1‐Pb’ 136.5(4), C1‐C2‐C3 176.9(6).

In Figure 3 the molecular structure of the dimeric lead acetylide **7** in the solid state is shown. Details of the structure analysis can be found in the Supporting Information. Lead acetylide compounds are rare and in the case of lead in oxidation state two only one acetylide coordination compound has been characterized by single crystal structure determination.^[15]^ [(Nacnac)Pb(C≡CPh)] {nacnac=HC(CMeNAr)_2_, Ar=2,6‐*i*Pr_2_C_6_H_3_} was synthesized by nucleophilic substitution and in the solid state the Pb−C interatomic distance is 2.276(3) Å and the Ph−C≡C−Pb triple bond 1.241(5) Å long.^[15]^ In compound **7** the phenylacetylide ligand is coordinated symmetrically by two lead atoms resulting in longer Pb−C distances of 2.467(5) and 2.498(5) Å (the acetylide ligands in the molecular structure of **7** exhibit a severe disorder and therefore bond lengths of the alkyne moiety are not discussed). Bridging acetylide coordination at two main group metals is a well‐known coordination mode in acetylide coordination chemistry.[^3^, ^16^] Aldridge and co‐workers treated diamidostannylene [(Me_3_SiDippN)_2_Sn] (Dipp=2,6‐ diisopropylphenyl) with phenylacetylene and isolated a dimeric alkynylstannylene with a bridging alkynyl ligand [(Me_3_SiDippNSn‐*μ*‐C≡CPh)_2_].^[3]^

The alkyne bridged dimer **7** shows in solution a resonance in the ^207^Pb NMR spectrum at 2734 ppm, in the range typical for triply coordinated low valent lead compounds.[^11b^, ^15a^, ^17^]

**Scheme 4:**
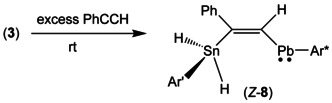
Addition of stannylplumbylene **3** to phenylacetylene.

Because phenylalkyne adds stannylplumbylene **3** at rt only slowly this reaction was carried out with 10 equiv of alkyne (Scheme 4). To avoid decomposition of the reaction product the reaction cannot be heated and has to be carried out at rt. After 15 minutes reaction time two products (*E*‐**8**, *Z*‐**8**) were found in the mixture. Both compounds were characterized by NMR spectroscopy (Table 1, series of spectra of conversion see Supporting Information).

**Table 1:** NMR signals of isomer *E*‐**8** and compound Z‐**8** after reaction of **3** with phenylacetylene.

	*E*‐**8**	*Z*‐**8**
^1^H NMR olefinic C*H* (ppm)	12.39 (s+d)	11.50 (s+d)
^3^ *J*_Sn‐H_ (Hz)	130	270
^1^H NMR Sn*H*_2_ (ppm)	4.46 (s + d)	4.17 (s+d)
^119^Sn NMR (ppm)	−444 (dt) (−20 °C)	−353 (dt)
^1^ *J*_119Sn‐H_ (Hz)	1827	1796

**Figure 4:**
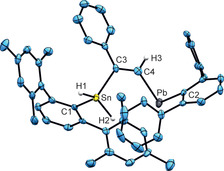
ORTEP of *Z*‐**8**. Hydrogen atoms were placed in idealized positions. *i*Pr groups and hydrogen atoms are omitted for the sake of clarity. Ellipsoids at 50 % probability. Interatomic distances [Å] and angles [deg]: C1‐Sn 2.161(7), C3‐Sn 2.146(8), C3‐C4 1.336(9), C2‐Pb 2.318(7), C4‐Pb 2.274(7), C1‐Sn‐C3 113.3(3), C4‐Pb‐C2 99.1(3).

**Scheme 5:**
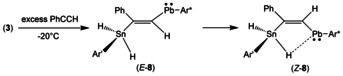
Formation of the kinetic and thermodynamic products.

After 1 h at rt only the final reaction product *Z*‐**8**, which was also characterized by single crystal structure analysis (Figure 4), was found in the NMR spectra. In order to characterize the intermediate *E*‐**8** and inhibit formation of the final product *Z*‐**8** we treated **3** at −20 °C with a high excess of PhC≡CH. After two minutes reaction time only formation of *E*‐**8**, which is the kinetically controlled reaction product, was detected in the ^1^H NMR spectrum. Because we found the signal for the C*H* unit of the isomer *E*‐**8** at high frequency (Table 1) comparable with the signal of the CH unit of the final product *Z*‐**8**, we assume formation of a C=CH‐Pb moiety in the intermediate *E*‐**8**. Furthermore, the ^119^Sn NMR signal of *E*‐**8** was found at −444 ppm and lies in the range of the signal found for *Z‐* **8** (−353 ppm). Therefore, we assign the intermediate as being the *trans*‐addition product *E*‐**8** (Scheme 5).

**Scheme 6:**
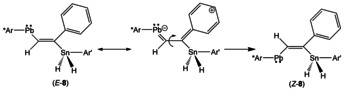
Isomerization of E‐**8** into *Z*‐**8**.

This assumption is supported by the difference of the ^3^ *J*_Sn‐H_ coupling constants: *E*‐**8** with *cis* ^3^ *J*_Sn‐H_ coupling 130 Hz and *Z*‐**8** with *trans* ^3^ *J*_Sn‐H_ coupling of 270 Hz. The *cis*‐stereochemistry of the CH unit and SnH_2_ moiety in the presumed isomer *E*‐**8** was verified by a NOESY experiment in which we detect a cross peak between the signals of the CH and SnH_2_ units (see Supporting Information Figure S20). After a couple of minutes at rt *E*‐**8** is completely isomerized into the *cis*‐addition product *Z*‐**8** (see Supporting Information Figure S19). Clearly, *Z*‐**8** is the thermodynamically controlled product. In the NBO analysis of *Z*‐**8** an intramolecular Pb–H–Sn interaction was found (see Supporting Information for Figure S25) between the empty p‐orbital of the lead atom and the Sn−H unit. This interaction could be responsible for the stabilization of *Z*‐**8**. However, in the room temperature ^1^H NMR spectrum of *Z*‐**8** only one signal for both protons of the SnH_2_ unit was found. Cooling a sample of *Z*‐**8** to −80 °C a broad signal for the SnH_2_ unit was detected and at −100 °C two signals were found indicating at rt a dynamic interplay between the SnH_2_ unit and the Pb atom (see Supporting Information Figure S15 for spectra). Due to the broadness of the signals and the short relaxation time of the lead atom ^207^Pb satellites could not be detected. The *trans*‐addition and formation of isomer *E*‐**8** could be due to the steric requirements found in starting material **3** (see Supporting Information Figure S1 for space filling drawing of **3**).^[5f]^ The bulky terphenyl substituents are arranged *anti* with respect to the Sn−Pb bond and therefore a presumed transition state of the addition reaction could point toward the *E*‐product. We propose for the possible *trans*–*cis* isomerization (Scheme 6) a delocalization of the double bond involving the empty p‐orbital at lead and the phenyl component.

To explain the higher reactivity of germylstannylene **1** in comparison to germylplumbylene **2** and stannylplumbylene **3** we assume as the first step of the alkyne addition a feasible interaction between electrophilic stannylene or plumbylene and nucleophilic alkyne. Descending Group 14 the stannylene **1** should exhibit higher electrophilicity and therefore higher reactivity in comparison to plumbylenes **2** and **3**.^[18]^

Formation of **4**, **5** and **8** are stereo‐ and regioselective reactions. In all three cases the phenyl substituent of the alkyne was found on the side of the smaller terphenyl substituent Ar’ due to reasons of stereochemistry. We discuss the *cis*‐selectivity of the addition products being a consequence of an intramolecular stabilization between the element hydride unit and the respective empty p‐orbital of the ylene, E–H–E’. This type of interaction which we discussed for compound *Z*‐**8** was also found for **4**. Deduced from low temperature ^1^H NMR spectra, in which a broadening of the GeH_2_ unit at −100 °C (see Supporting Information Figure S3) was found and NLMO analysis exhibiting a Ge–H–Sn interaction, this interaction might be an common intramolecular interaction in the series of addition products **4**, **5** and **8**.

The result of the crystal structure analysis of *Z*‐**8** is shown in Figure 4. As already discussed for compound **5**, compound *Z*‐**8** is also an aryl vinyl plumbylene and bond lengths could be compared with a small number of cyclic molecules. In these compounds the Pb−C(vinyl) distances are 2.329(3), 2.306(5), 2.320(5) Å, and Pb−C(aryl) distance 2.360(3) Å is slightly longer in comparison to molecule *Z*‐**8** Pb−C(vinyl) 2.274(7) Å, Pb−C(aryl) 2.318(7) Å.^[10]^

Alkyne addition product *Z*‐**8** was also characterized by NMR spectroscopy. The ^1^H and ^119^Sn NMR signals of the SnH_2_ unit were found at 4.17 ppm and −353 ppm and can be compared with the signals found for the SnH_2_ unit in the alkyne addition product [Ar*SnCHCPhSnH_2_Ar*] synthesized in reaction between the dimeric tin hydride [(Ar*SnH)_2_] and phenylalkyne (5.05 ppm, −324 ppm).^[2i]^ The signal which was found in the ^207^Pb NMR spectrum for plumbylene *Z*‐**8** (6436 ppm) is indicative of a monomeric plumbylene and lies in the range of signals found for plumbylenes **2** (9183 ppm) and **3** (8885 ppm) (see Scheme 1 for **2** and **3**).[^5f^, ^11^]

In the ^1^H and ^13^C NMR spectra of *Z*‐**8** the signals of the Pb−*CH=C* unit were found in the ^1^H NMR spectrum at 11.50 ppm and in the ^13^C{^1^H} NMR spectrum at 284.7 ppm. To rationalize these high frequency signals relativistic DFT calculations of compound *Z*‐**8** were performed.^[12]^ Based on the molecular structure determined in the solid state the structure of *Z*‐**8** was optimized at DFT level with scalar relativistic effective core potentials. Isotropic ^13^C and ^1^H NMR shifts were then calculated at the level of the two‐component zero‐order regular approximation (SO‐ZORA) using the ADF program,^[19]^ as well as the four‐component matrix Dirac–Kohn–Sham (mDKS) level using the ReSpect program package^[20]^ (Table 2; notably, both approaches included the exchange‐correlation kernel and used gauge‐including atomic orbitals, GIAOs). Both relativistic levels account for the important SO‐HALA effects (see above) and thus reproduce the experimentally observed high‐frequency ^1^H and ^13^C shifts of this unit. In order to identify the orbitals responsible for variations in the chemical shift, the SO‐ZORA computations were broken down into natural bond orbitals (NBOs) and natural localized molecular orbitals (NLMOs).^[21]^ The isotropic shielding of C4 is dominated by the NLMO representing the σ_p_‐bond between Pb−C4 (contribution −223.9 ppm), the σ‐C4−H NLMO (−56.6 ppm) and the π_p_‐C3−C4 NLMO (−56.3 ppm) which affect the paramagnetic and spin–orbit parts of the isotropic shielding (see Supporting Information Figures S25–30 for NLMOs).

**Table 2:** Results of NMR calculations of compound *Z*‐**8** (*δ* in ppm).

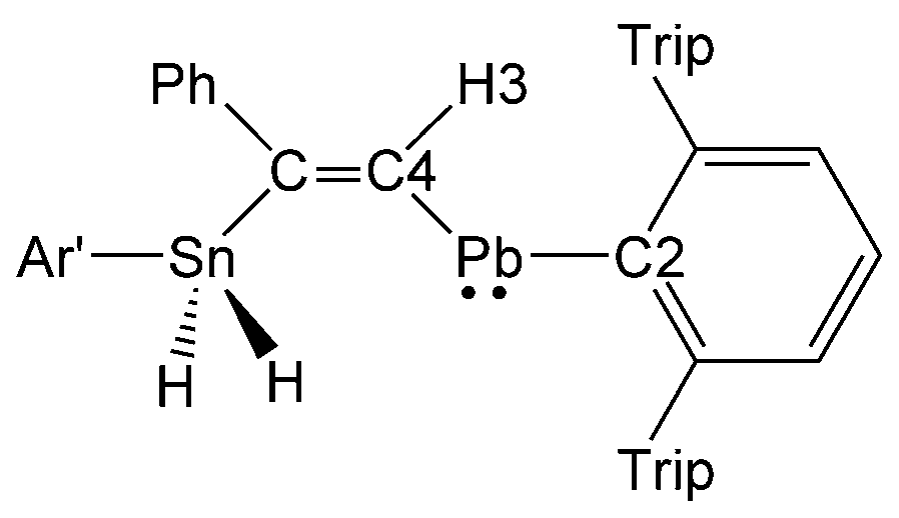			
	Exp.	ADF SO‐ZORA^[a]^	ReSpect 4c‐mDKS^[b]^
C2	259.6	258.0	250.1
C4	284.7	298.6	293.0
H3	11.50	11.0	10.1

Basis sets: [a] Sn, Pb, C, TZ2P; H, TZP. [b] Sn, Pb, Dyall‐VTZ; C, H, uncontracted IGLO‐II.

In the case of the high frequency shift of the olefinic proton H3 (Figure 4) a large shielding contribution of −4.3 ppm comes from the NLMO representing the σ_p_‐Pb−C4 bond. Further shielding contributions are −2.0 ppm from σ‐C4−H, −1.8 from π_p_‐C3−C4, −1.4 from σ‐Sn−C3 and −1.2 ppm from the Pb lone pair. Furthermore, part of the aryl moiety of the Pb‐terphenyl substituent neighboring the olefinic CH unit contributes +1.47 ppm to the shielding.

The addition product **5** exhibits also high frequency shifts for the proton of the olefinic Ge−*CH*=CPh unit (^1^H NMR 13.10 ppm) and for the lead‐bound carbon atoms (^13^C NMR 273.6 ppm for *C*(Ph)−Pb, 263.1 ppm Pb−*C* ${}_{\mathrm{Ph}\left(\mathrm{Mes}\right){}_{2}}$ ). The role of the SO effects on the NMR shifts has been documented by calculating the NMR shifts at scalar‐relativistic ZORA level, i.e. without SO contributions. These computations do not agree with the experiment and provide shifts at much too low frequencies. This shows the dominant importance of SO‐HALA effects (Table S5 in the Supporting Information).

**Scheme 7:**
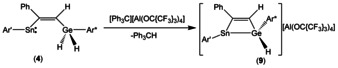
Hydride abstraction from alkyne addition product **4**.

To study a feasible hydride abstraction of the diorganogermanium dihydride **4** the alkyne addition product was reacted with one equivalent of the trityl salt [Ph_3_C][Al(OC{CF_3_}_3_)_4_] (Scheme 7).^[22]^ A weakly coordinating anion was used because highly reactive triorganogermylium cations reported in the literature were stabilized by weakly coordinating anions such as tetrakis(pentafluorophenyl)borate, permethylated carba‐*closo*‐dodecaborate or tetra(perfluoro‐*tert*‐butoxy)aluminate.^[23]^ The hydride abstraction was carried out in a 1:2 mixture between 1,2‐difluorobenzene and benzene as solvent at room temperature (Scheme 7). Based on the ^1^H NMR spectrum the yield of **9** is roughly 70 % and another undefined species was found in the spectrum. Crystallization from 1,2‐difluorobenzene/hexane resulted in a very small amount of crystals of salt **9** which are suitable for single crystal diffraction.

**Figure 5:**
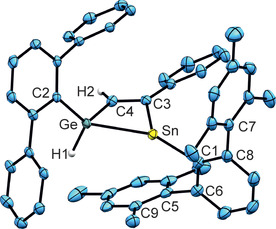
ORTEP of **9**. Hydrogen atom connected to the germanium atom was found. Other hydrogen atoms, which are not shown were placed in idealized positions. *i*Pr groups and anion [Al(OC{CF_3_}_3_)_4_]^−^ are omitted for the sake of clarity. Ellipsoids at 50 % probability. Interatomic distances [Å] and angles [deg]: Ge‐Sn 2.669(1), C1‐Sn 2.145(2), C3‐Sn 2.138(2), C3‐C4 1.342(3), C2‐Ge 1.942(2), C4‐Ge 1.949(2), Sn‐C5 2.765(2), Sn‐C9 3.054(2), C1‐Sn‐C3 123.3(1), C4‐Ge‐C2 120.4(1), C4‐Ge‐Sn 70.6(1), C3‐Sn‐Ge 70.8(1), C1‐Sn‐Ge 157.9(1), C3‐C4‐Ge 116.6(2), C4‐C3‐Sn 100.9(2), Sn‐C1‐C6 106.5(2), Sn‐C1‐C8 131.3(2).

The molecular structure of the cation of **9** is depicted in Figure 5 together with selected interatomic distances and angles. The most striking feature is the formation of a donor–acceptor bond between the stannylene tin atom and the germylium cation which results in the formation of a triply coordinated stannylium cation.

Based on the solid state structure the structure of cation **9** was optimized using DFT calculations and the bonding was analyzed using NLMOs.^[24]^ Forty four percent of the tin lone pair is donated into the empty orbital of the germanium atom (see Supporting Information Figure S32) which supports the interpretation of the formation of a stannylium cation. Stannylium cations [R_3_Sn]^+^ stabilized by weakly coordinating anions were intensively investigated and ^119^Sn NMR signals documented (R=Me, 348 ppm; *n*Bu, 454 ppm, Mes, 233 ppm; Trip, 714 ppm; Dur, 720 ppm; *t*Bu_2_MeSi, 2653 ppm).[^23d^, ^23e^, ^23h^, ^25^] Cation **9** exhibits an ^119^Sn NMR signal at 429 ppm. The ^119^Sn NMR chemical shift of 404 ppm computed at SO‐ZORA level is close to the experimentally determined shift of 429 ppm. In the solid state the tin atom exhibits contacts to C5 and C9 which are manifested by a decrease of the angle Sn‐C1‐C6 to 106.5(2)° and interatomic distances of Sn−C5 2.765(2), Sn−C9 3.054(2) Å. This type of interaction was also discussed for the cation [Ar*Sn(C_6_H_6_)]^+^.^[26]^

To the best of our knowledge and based on a search in the Cambridge structural database a germastannacyclobutene ring is so far unknown. However, examples for the silagermete ring molecule were already published in the literature.[^2k^, ^2o^, ^27^] The Sn−Ge bond length of 2.669(1) Å is comparable to the Sn‐Ge bond length found in **1** [2.6686(3) Å] and slightly shorter than distances reported for a germylenestannylene [2.721(1) Å] or germylstannylenes [2.722(1), 2.746(1) Å].^[28]^ The tin atom exhibits a nearly trigonal planar coordination mode (sum of angles=352°) and the germanium atom a tetrahedral arrangement. Upon ring formation the angles Sn‐C3‐C4 (24°) and C3‐C4‐Ge decrease by 10° in comparison to the starting material **4**. In the ^1^H NMR spectrum the Ge−H hydride shows a signal at 7.27 ppm with tin satellites (^2^ *J*_Sn‐H_=551 Hz) indicating a ring structure also in solution. The starting material **4** shows a signal for the GeH_2_ unit at 3.76 ppm. The signal for the vinyl proton was found at slightly lower frequency (6.17 ppm) in comparison to starting material **4** and shows tin satellites (^3^ *J*_Sn‐H_ 93 Hz).

## Conclusions

Tetryl‐tetrylenes react with phenylacetylene under regio‐ and stereoselective 1,2‐addition. Germylstannylene reacts with one equivalent alkyne at rt to yield the addition product. Germylplumbylene reacts with an excess of alkyne and the product exhibits a further reaction with excess alkyne to yield a dimeric lead acetylide compound. In the case of stannylplumbylene, formation of a kinetically controlled *trans*‐addition product was found which isomerizes at rt to give the thermodynamically more stable *cis*‐isomer showing an intramolecular Sn–H–Pb interaction. Hydride abstraction was studied in the case of the alkyne addition product [Ar*Sn‐(CH=PhC)‐GeH_2_Ar] in the reaction with the trityl salt [Ph_3_C][Al(OC{CF_3_}_3_)_4_]. A hydride substituent was abstracted from the germanium atom and in the cationic product a bond between the stannylene tin atom and the cationic germanium atom was formed. Clearly, the stannylene has reacted as a Lewis base with the Lewis acidic germylium cation. The reaction sequence: element bond formation, alkyne addition and hydride abstraction is a synthetic pathway for the formation of a Ge−Sn cyclobutene ring. Spin–orbit‐induced heavy atom effects are apparent for the NMR shifts of atoms directly bound to the heavier group 14 atoms, but also for atoms removed by three bonds in many of the species observed. They lead to distinct high frequency shifts, as has been confirmed by relativistic DFT calculations.

## Experimental Section

All manipulations were carried out under an argon atmosphere using standard Schlenk techniques and gloveboxes. *n*‐Pentane and *n*‐hexane were dried using a M. Braun Solvent Purification System (SPS). All other solvents were distilled from sodium or sodium/potassium alloy. All solvents were subsequently degassed by 3× freeze/pump/thaw. [2,6‐Mes_2_C_6_H_3_SnCl]_2_, [2,6‐Trip_2_C_6_H_3_SnCl]_2_, [2,6‐Trip_2_C_6_H_3_GeCl]_2_, [2,6‐Trip_2_C_6_H_3_PbBr]_2,_ [2,6‐Mes_2_C_6_H_3_PbBr]_2_, [2,6‐Trip_2_C_6_H_3_PbH]_2_ and [Ph_3_C][Al(OC{CF_3_}_3_)_4_] were synthesized following literature procedures.[^22a^, ^29^] Further chemicals were purchased commercially and used as received. Elemental analyses were performed at the Institute of Inorganic Chemistry, University of Tübingen using a Vario MICRO EL analyzer.

NMR spectra were recorded on a Bruker DRX‐250 NMR spectrometer (^1^H, 250.13 MHz; ^13^C, 62.90 MHz; ^119^Sn, 93.28 MHz, ^207^Pb, 52.29 Hz) equipped with a 5 mm TBO probe head, a Bruker AvanceII+400 NMR spectrometer (^1^H, 400.11 MHz; ^13^C, 100.61 MHz) equipped with a 5 mm QNP (quad nucleus probe) head and a Bruker AvanceII+500 NMR‐spectrometer (^1^H, 500.13 MHz; ^13^C, 125.76 MHz; ^119^Sn, 186.50 MHz) equipped with a 5 mm TBO probe head and a setup for variable temperature. The chemical shifts are reported in *δ* values in ppm relative to external SiMe_4_ (^1^H, ^13^C), SnMe_4_ (^119^Sn) or PbMe_4_ (^207^Pb) using the chemical shift of the solvent ^2^H resonance frequency and *Ξ=*25.145020 % for ^13^C, *Ξ=*37.290632 % for ^119^Sn and *Ξ=*20.920599 % for ^207^Pb.^[30]^ The multiplicity of the signals is abbreviated as s=singlet, d=doublet, t=triplet, quint=quintet, sept=septet and m=multiplet or unresolved. The proton and carbon signals were assigned by detailed analysis of ^1^H, ^13^C{^1^H}, ^1^H‐^1^H COSY, ^1^H‐^13^C HSQC, ^1^H‐^13^C HMBC and ^13^C{^1^H} DEPT‐135 spectra. For variable temperature measurements the sample temperature was stabilized with a Bruker BVT 3200 temperature controller. The temperatures given are uncorrected.

X‐ray data were collected with a Bruker Smart APEX II diffractometer with graphite‐monochromated Mo Kα radiation or a Bruker APEX II Duo diffractometer with a Mo IμS microfocus tube and TRIUMPH monochromator. The programs used were Bruker's APEX2 v2011.8‐0, including SADABS for absorption correction, SAINT for data reduction and SHELXS for structure solution, as well as the WinGX suite of programs version 1.70.01 or the GUI ShelXle, including SHELXL for structure refinement.^[31]^

DFT calculations of **5**, *Z*‐**8** and **9** were carried out with Gaussian09,^[32]^ the ADF^[19a]^ and Respect^[20a]^ program packages were used for shielding calculations and the NBO6.0^[21e]^ software was used to obtain natural bond orbitals and for analyses of shielding data (for further details see the Supporting Information).

### Synthesis and characterization

Synthesis of Ar*GeH_2_C(H)=C(Ph)SnAr’ (4): A solution of phenylacetylene (5.8 mg, 0.056 mmol, 1.1 equiv) in toluene (1 mL) was added to a solution of Ar*GeH_2_‐SnAr’ (50 mg, 0.051 mmol, 1 equiv) in toluene (1.5 mL). The color changes immediately from violet to pink. After stirring for 10 minutes at room temperature all volatiles were evaporated under reduced pressure to yield Ar*GeH_2_CHC(Ph)SnAr’ as pink powder (55.1 mg, 0.051, 99 %). Crystals suitable for X‐ray diffraction were obtained from saturated solutions of hexane at −40 °C. Analytical data:^1^H NMR (500.13 MHz, C_6_D_6_): *δ=*7.16 (t overlayed with solvent signal, 1 H, ^3^ *J*_H‐H_=7.5 Hz, *p*‐C_6_ *H_3_*(Mes)_2_), 7.11–7.07 (m, 2 H, *m*‐C_6_ *H_5_*), 7.10 (s, 4 H, (C_6_ *H_2_)*_Trip_), 7.09 (s, 3 H, C_6_ *H_3_*(Trip)_2_), 6.96 (m, 1 H, *p*‐C_6_ *H_5_*), 6.90 (d, 2 H, ^3^ *J*_H‐H_=7.4 Hz, *m*‐C_6_ *H_3_*(Mes)_2_), 6.79 (t, 1 H, ^3^ *J*_H‐H_=5.1 Hz, Ge‐C*H*), 6.75 (s, 4 H, (C_6_ *H_2_)*_Mes_), 6.51 (m, 2 H, *o*‐C_6_ *H_5_*), 3.76 (d, 2 H, ^3^ *J*_H‐H_=5.1 Hz, Ge*H_2_*), 2.88–2.77 (m, 6 H, *o*/*p*‐C*H*Me_2_), 2.20 (s, 6 H, *p‐Me*), 1.98 (s, 12 H, *o‐Me*), 1.27 (d, 12 H, ^3^ *J*_H‐H_=6.8 Hz, *o*‐CH*Me*_2_), 1.27 (d, 12 H, ^3^ *J*_H‐H_=6.9 Hz, *p*‐CH*Me*_2_), 1.05 ppm (d, 12 H, ^3^ *J*_H‐H_=6.8 Hz, *o*‐CH*Me*_2_). ^13^C{^1^H} NMR (125.76 MHz, C_6_D_6_): *δ=*206.7 (s, *C*(Ph)‐Sn), 180.4 (s, *C_C6H3_*‐Sn), 149.8 (s, *C*_C6H3_‐Ge), 148.2 (s, *p‐C*_Trip_), 147.2 (s, *o‐C*_*Ph*(Trip)2_), 146.9 (s, *o‐C*_*Ph*(Mes)2_), 146.2 (s, *o‐C*_Trip_), 138.7 (s, *ipso*‐*C*_Mes_), 137.8 (s, *ipso‐C*_Trip_), 136.6 (s, *ipso‐C_6_*H_5_), 136.5 (s, Ge‐*C*H), 136.4 (s, *p‐C*_Mes_), 136.2 (s, *o‐C*_Mes_), 129.3 (s, *m‐C_6_*H_5_), 128.9 (s, *m‐C*_Mes_), 128.6 (s, *m‐C*_*Ph*(Mes)2_), 128.3 (s, *p‐C*_*Ph*(Mes)2_), 127.8 (s, *m‐C*_*Ph*(Trip)2_), 127.7 (s, *p‐C*_*Ph*(Trip)2_), 125.6 (s, *p‐C_6_*H_5_), 125.0 (s, *o‐C_6_*H_5_), 120.7 (s, *m‐C*_Trip_), 34.3 (s, *p‐C*HMe_2_), 30.6 (s, *o‐C*HMe_2_), 25.5 (s, *p*‐CH*Me_2_*), 24.0 (s, o/*p*‐CH*Me_2_*), 22.9 (s, o/*p*‐CH*Me_2_*), 21.2 (s, *o‐Me*), 21.1 ppm (s, *p‐Me*). ^119^Sn NMR (186.50 MHz, C_6_D_6_): *δ=*1630 ppm (s, *Sn*). Anal. calcd for C_68_H_82_GeSn: C 74.88, H 7.58. Found: C 74.72, H 7.54.

Synthesis of Ar*GeH_2_C(H)=C(Ph)PbAr’ (5), Ar*GeH_2_C(H)C(H)(Ph) (6), [Ar'PbC≡C(Ph)]_2_ (7): A solution of phenylacetylene (29.0 mg, 0.279 mmol, 15 equiv) in benzene (0.25 mL) was added to a solution of Ar*GeH_2_‐PbAr’ (20 mg, 0.018 mmol, 1 equiv) in benzene (0.25 mL). The color changed after 5 hours from blue to violet. All volatiles were evaporated under reduced pressure and the residue was dissolved in hexane and stored at −40 °C to yield a small amount of temperature sensitive Ar*GeH_2_C(H)=C(Ph)PbAr’ (**5**) as violet crystals suitable for X‐ray diffraction. Because of the very small amounts of isolated compound **5** a determination of an elemental analysis was impossible. Without interrupting the reaction and stirring the original benzene reaction mixture overnight, the color changes from violet to brown. All volatiles were evaporated under reduced pressure, the brown residue was dissolved in hexane, concentrated, and stored at −40 °C. Yellow crystals were isolated and characterized: [Ar'PbC≡C(Ph)]_2_ (**7**) (3.8 mg, 0.009 mmol, 33 %). From the mother liquor among other unidentified compounds Ar*GeH_2_C(H)=C(H)(Ph) (**6**) was characterized.

Ar*GeH_2_C(H)=C(Ph)PbAr’ (5): ^1^H NMR (500.13 MHz, C_6_D_6_, 298.2 K): *δ=*13.10 (t, 1 H, *H*C=, ^3^ *J*_HH_=4.9 Hz,), 7.50 (d, 2 H, ^3^ *J*_HH_=7.5 Hz, *m*‐C_6_ *H_3_*(Mes)_2_), 7.32–7.24 (m, 4 H, *p*‐C_6_ *H_3_*(Trip)_2_, *p*‐C_6_ *H_3_*(Mes)_2_, *o*‐C_6_ *H*_5_), 7.11 (s, 4 H, (C_6_ *H*_2_)_Trip_), 7.09 (m, 3 H, *m*/*p*‐C_6_ *H*_5_), 6.77 (s, 4 H, (C_6_ *H*_2_)_Mes_), 6.44 (m, 2 H, *m*‐C_6_ *H_3_*(Trip)_2_), 3.71 (d, 2 H, ^3^ *J*_HH_=4.9 Hz, Ge*H*_2_), 2.83 (m, 6 H, *p*‐C*H*Me_2_, *o*‐C*H*Me_2_), 2.20 (s, 6 H, *p‐Me*), 2.02 (s, 12 H, *o‐Me*), 1.28 (d, 12 H, ^3^ *J*_HH_=6.8 Hz, *p*‐CH*Me*_2_), 1.25 (d, 12 H, ^3^ *J*_HH_=7.1 Hz, *o*‐CH*Me*_2_), 1.05 ppm (d, 12 H, ^3^ *J*_HH_=7.1 Hz, *o*‐CH*Me*_2_). ^13^C{^1^H} NMR (125.76 MHz, C_6_D_6_, 280.15 K): *δ=*273.6 (s, *C*(Ph)‐Pb), 263.1 (s, Pb*‐C*_*Ph*(Mes)2_), 157.5 (*ipso‐C_6_*H_5_), 148.5(s, *p‐C*_Trip_), 148.2 (s, *o‐C*_*Ph*(Mes)2_), 147.2 (s, Ge‐*C*_*Ph*(Trip)2_), 146.3 (s, *o‐C*_Trip_), 146.1 (s, *o‐C*_*Ph*(Trip)2_), 138.9 (s, *p‐C*_Mes_), 138.6 (s, *ipso*‐*C*_Mes_), 138.1 (s, *ipso*‐*C*_Trip_), 136.4 (s, *o‐C*_Mes_), 135.3 (s, *m‐C*_*Ph*(Mes)2_), 135.1 (*C*(H)‐Ge), 129.6 (s, *p‐C*_*Ph*(Mes)2_), 129.0 (s, *p‐C*_*Ph*(Trip)2_), 128.8 (*m‐C*_Mes_), 127.3(s, *m‐C*_*Ph*(Trip)2_), 128.0 (*o‐C_6_*H_5_), 126.9(s, *m‐C_6_*H_5_), 126.4(s, *p‐C_6_*H_5_), 120.7 (s, *m‐C*_Trip_), 34.4 (s, *p‐C*HMe_2_), 30.7 (s, *o‐C*HMe_2_), 25.6 (s, *o*‐CH*Me_2_*), 24.2 (s, *p*‐CH*Me_2_*), 22.9 (s, *o*‐CH*Me_2_*), 21.2 ppm (s, *o‐Me*, *p‐Me*). ^207^Pb{^1^H} (104.63 MHz, C_6_D_6_, 280.15 K): *δ*=7591 ppm (s, *Pb*).

Ar*GeH_2_C(H)=C(H)(Ph) (6): ^1^H NMR (500.13 MHz, 298.2 K, C_6_D_6_): *δ=*7.24–7.08 (m, 12 H, *m*‐C*H*_Trip_, *m*‐C*H*_TripPh_, *p*‐C*H*_TripPh_, *m*‐C*H*_Ph_, *p*‐C*H*_Ph_, *o*‐C*H*_Ph_), 6.60 (d, 1 H, C*H*CHPh, ^3^ *J*_HH_=18.4 Hz), 5.40 (dt, 1 H, C*H*CHPh, ^3^ *J*_HH_=18.4 Hz, ^3^ *J*_HH_=4.4 Hz), 4.36 (d, 2 H, Ge*H*_2_, ^3^ *J*_HH_=4.4 Hz), 2.94 (sept, 4 H, *o*‐C*H*(CH_3_)_2_), 2.89 (sept, 2 H, *p*‐C*H*(CH_3_)_2_, 1.32 (d, 12 H, *o*‐CH(C*H*_3_)_2_, ^3^ *J*_HH_=6.9 Hz), 1.30 (d, 12 H, *p*‐CH(C*H*_3_)_2_), ^3^ *J*_HH_=6.9 Hz), 1.13 ppm (d, 12 H, *o*‐CH(C*H*_3_)_2_, ^3^ *J*_HH_=6.9 Hz).^13^C{^1^H} NMR (125.76 MHz, 298.2 K, C_6_D_6_): *δ=*148.5 (*p‐C*_Trip_), 147.2 (Ge‐*C*_TripPh_), 146.5 (*o‐C*_Trip_), 146.3 (*o‐C*_TripPh_), 144.6 (CH*C*HPh), 137.9 (*i*‐*C_Ph_*), 137.6 (*i‐C*_Trip_), 128.7 (*m‐C*_TripPh_), 128.2 (*p‐C*_TripPh_), 127.9 (*m‐C_Ph_*), 127.7 (*p‐C_Ph_*), 126.3 (*o‐C_Ph_*), 123.7 (*C*HCHPh), 120.7 (*m‐C*_Trip_), 34.4 (*p‐C*H(CH_3_)_2_), 30.8 (*o‐C*H(CH_3_)_2_), 25.5 (*o*‐CH(*C*H_3_)_2_), 22.8 (*p*‐CH(*C*H_3_)_2_), 22.6 ppm (*o*‐CH(*C*H_3_)_2_).

[**Ar'PbC≡C(Ph)]_2_ (7)**: ^1^H NMR (500.13 MHz, 298.2 K, C_6_D_6_): *δ*=7.40–6.90 (m, 8 H, *m*‐CH_Mes_, *p*‐CH_Mes_, CH_Ph_), 6.81 (s, 4 H, *m*‐CH_Mes_), 2.26 (s, 6 H, *p*‐C*Me*_2_), 2.15 ppm (s, 12 H, *o*‐Me). ^13^C{^1^H} NMR (125.76 MHz, 298.2 K, C_6_D_6_): *δ=*150.2 (Pb‐C*C*Ph), 141.4 (*o*‐C_PhMes_), 139.6. (*ipso*‐C_Mes_), 136.9 (*o‐C*_Mes_), 135.9 (*p‐C*_Mes_), 131.9 (*ipso*‐*C_Ph_*), 131.7 (*m*‐C_PhMes_), 131.1 (*o‐C_Ph_*), 128.3 (*m‐C*_Mes_), 127.3 (*m‐C_Ph_*), 126.1 (*p‐C_Ph_*), 124.3 (*p‐C*_PhMes_), 21.5 (*o‐C*H_3_), 20.8 ppm (*p‐C*H_3_). (Pb‐*C*CPh), and (*i*‐C_PhMes_), signals could not be detected. ^207^Pb NMR (52.33 MHz, 298.2 K, C_6_D_6_): *δ=*2734 ppm (s).

Synthesis of Ar'SnH_2_C(Ph)=C(H)PbAr* (*Z*‐8): A solution of phenylacetylene (46 mg, 0.45 mmol, 10 equiv) in toluene (1 mL) was added to a solution of Ar'SnH_2_‐PbAr* (50 mg, 0.045 mmol, 1 equiv) in toluene (2 mL). The reaction mixture was stirred for four hours at room temperature while the color changed from blue to violet. All volatiles were evaporated under reduced. The residue was dissolved in hexane (1 mL) filtered through a syringe filter and stored for several days at −40 °C to yield Ar'SnH_2_C(Ph)CHPbAr* as red crystals (13 mg, 0.011 mmol, 24 %). Compound **8** shows decomposition at rt in solution. Because formation of **8** with one equivalent phenylacetylene is slow 10 equiv were used. Due to the thermal sensitivity of **8** in solution NMR spectra were obtained at −10 °C. Analytical data: ^1^H NMR (500.13 MHz, C_6_D_6_, 263.15 K): *δ=*11.50 ppm (t + satellites, 1 H, ^4^ *J*_H‐H_=2.3 Hz, ^3^ *J*_119Sn‐H_=ca. 270 Hz, C*H*‐Pb), 7.71 (d, 2 H, ^3^J_H‐H_=7.5 Hz, *m*‐C_6_ *H_3_*(Trip)_2_), 7.33 (t, 1 H, ^3^ *J*_H‐H_=7.5 Hz, *p*‐C_6_ *H_3_*(Trip)_2_), 7.21 (t, 3 H, ^3^ *J*_H‐H_=7.72 Hz, *o*‐C_6_ *H*_5_), 7.15 (s, 4 H, (C_6_ *H*_2_)_Trip_), 7.09 (t, 1 H, ^3^ *J*_H‐H_=7.6 Hz, *p*‐C_6_ *H_3_*(Mes)_2_), 7.04 (m, 3 H, *m*/*p*‐C_6_ *H*_5_), 6.84 (d, 2 H, ^3^ *J*_H‐H_=7.6 Hz, *m*‐C_6_ *H_3_*(Mes)_2_), 6.77 (s, 4 H, (C_6_ *H*_2_)_Mes_), 4.17 (d + satellites, 2 H, ^4^ *J*_H‐H_=2.3 Hz, ^1^ *J*_*119Sn*‐H_=1796 Hz, Sn*H*_2_), 3.22 (sept, 4 H, ^3^ *J*_H‐H_=7.1 Hz, *o*‐C*H*Me_2_), 2.87 (sept, 2 H, ^3^ *J*_H‐H_=6.8 Hz, *p*‐C*H*Me_2_), 2.17 (s, 6 H, *p‐Me*), 1.91 (s, 12 H, *o‐Me*), 1.31 (d, 12 H, ^3^ *J*_H‐H_=6.8 Hz, *p*‐CH*Me*_2_), 1.29 (d, 12 H, ^3^ *J*_H‐H_=7.1 Hz, *o*‐CH*Me*_2_), 1.08 (d, 12 H, ^3^ *J*_H‐H_=7.1 Hz, *o*‐CH*Me*_2_). ^13^C{^1^H} NMR (125.76 MHz, C_6_D_6_, 263.15 K): *δ=*284.7 (s, Pb‐*C*H), 259.6 (s, *C_C6H3_*‐Pb), 161.3 (*ipso‐C_6_*H_5_), 157.3 (s, *C*(Ph)‐Sn), 150.0 (s, *o‐C*_*Ph*(Mes)2_), 148.1 (s, *p‐C*_Trip_), 146.8 (s, *o‐C*_Trip_), 146.1 (s, *o‐C*_*Ph*(Trip)2_), 141.2 (s, *ipso*‐*C*_Mes_), 138.4 (s, *C_C6H3_*‐Sn), 136.6 (s, *p‐C*_Mes_), 136.2 (s, *m‐C*_*Ph*(Mes)2_), 135.9 (s, *o‐C*_Mes_), 135.6 (s, *ipso*‐*C*_Trip_), 129.9 (s, *p‐C*_*Ph*(Trip)2_), 128.5 (s, *m‐C*_Mes_), 127.9 (s overlayed with solvent signal, *o‐C_6_*H_5_), 127.2 (s, *m‐C*_*Ph*(Trip)2_), 126.3 (s, *m‐C_6_*H_5_), 126.2 (s, *p‐C_6_*H_5_), 124.5 (s, *p‐C*_*Ph*(Mes)2_), 121.3 (s, *m‐C*_Trip_), 34.3 (s, *p‐C*HMe_2_), 30.6 (s, *o‐C*HMe_2_), 26.1 (s, *o*‐CH*Me_2_*), 24.2 (s, *p*‐CH*Me_2_*), 23.5 (s, *o*‐CH*Me_2_*), 21.2 (s, *p‐Me*), 20.9 ppm (s, *o‐Me*). ^119^Sn{^1^H} NMR (186.50 MHz, C_6_D_6_, 263.15 K): *δ=*−353 ppm (s, *Sn*). ^207^Pb{^1^H} NMR (104.63 MHz, C_6_D_6_, 263.15 K): *δ=*6436 ppm (s, *Pb*). Anal. calcd for C_68_H_82_PbSn: C 66.66, H 6.75. Found: C 66.99, H 6.70.

Synthesis of [Ar*GeHC(H)=C(Ph)SnAr’][Al(OC(CF_3_)_3_)_4_] (9): [Ph_3_C][Al(OC(CF_3_)_3_)_4_] (27.7 mg, 0.023 mmol, 1 equiv) was dissolved in a mixture of 1,2‐difluorobenzene (0.2 mL) and benzene (0.4 mL) and added to a solution of Ar*GeH_2_CHC(Ph)SnAr’ (25 mg, 0.023 mmol, 1 equiv) in benzene (0.4 mL). After addition the color changed to red. The mixture was stirred for four hours at room temperature. Volatiles were removed under reduced pressure and the red oily residue was washed with hexane (3× 0.5 mL) to remove the formed triphenylmethane. The crude product was dried in vacuo and dissolved in 1,2‐difluorobenzene (0.1 mL). Hexane was allowed to diffuse into the reaction mixture over a period of three weeks at room temperature. The supernatant solution was decanted and the red crystals were washed with cold pentane (2× 0.1 mL) and dried in vacuo to yield [Ar*GeHCHC(Ph)SnAr’][Al(OC(CF_3_)_3_)_4_]. Analytical data: ^1^H NMR (500.13 MHz, 0.3 mL C_6_D_6_+0.1 mL C_6_H_4_F_2_): *δ=*7.27 (s + satellites, 1 H, ^2^ *J*_119Sn‐H_=551 Hz, Ge*H*), 7.26–7.23 (m, Aryl‐*H*s), 7.05–7.01 (m, Aryl‐*H*s), 6.84 (m, Aryl‐*H*s), 6.17 (s + satellites, ^3^ *J*_Sn‐H_=93 Hz, Ge‐C*H*), 2.68 (br m, 2 H, *p*‐C*H*Me_2_), 2.50 (br sept, 4 H, ^3^ *J*_H‐H_=6.9 Hz, *o*‐C*H*Me_2_), 2.15 (s, 6 H, *p‐Me*), 1.84 (s, 12 H, *o‐Me*), 1.01 (d, 12 H, ^3^ *J*_H‐H_=6.8 Hz, *p*‐CH*Me*_2_), 0.96 (d, 12 H, ^3^ *J*_H‐H_=6.9 Hz, *o*‐CH*Me*_2_), 0.89 ppm (d, 12 H, ^3^ *J*_H‐H_=6.9 Hz, *o*‐CH*Me*_2_). ^13^C{^1^H} NMR (125.76 MHz, 0.3 mL C_6_D_6_ + 0.1 mL C_6_H_4_F_2_): *δ=*187.2 (*C*‐Sn), 146.9 (*p‐C*_Trip_), 146.2 (*o‐C*_Trip_), 140.2 (*p‐C*_Mes_), 135.5 (Ge‐*C*H), 135.9 (*ipso*‐*C*_Mes_), 134.2 (*o‐C*_Mes_), 130.8 (*m‐C*_Mes_), 33.8 (*p‐C*HMe_2_), 30.7 (*o‐C*HMe_2_), 24.1 (*p*‐CH*Me*_2_), 23.7 (*o*‐CH*Me*_2_), 20.3 (*o‐Me*), 20.1 ppm (*p‐Me*). ^119^Sn NMR (186.50 MHz, 0.3 mL C_6_D_6_+0.1 mL C_6_H_4_F_2_): *δ=*429 ppm (d, ^2^ *J*_119Sn‐H_=551 Hz, *Sn*).

Deposition Numbers 2046116, 2046112, 2046113, 2046115, and 2046114 contain the supplementary crystallographic data for this paper. These data are provided free of charge by the joint Cambridge Crystallographic Data Centre and Fachinformationszentrum Karlsruhe Access Structures service www.ccdc.cam.ac.uk/structures.

## Conflict of interest

The authors declare no conflict of interest.

## Supporting information

As a service to our authors and readers, this journal provides supporting information supplied by the authors. Such materials are peer reviewed and may be re‐organized for online delivery, but are not copy‐edited or typeset. Technical support issues arising from supporting information (other than missing files) should be addressed to the authors.

Supplementary
