# Supplementary material for: Tetryl‐Tetrylene Addition to Phenylacetylene
Source: Chemistry. 2021 Feb 4;27(14):4691–9. doi: 10.1002/chem.202005119 (PMC7986144; doi:10.1002/chem.202005119)
Supplement: Supplementary file 1 — Supplementary [file CHEM-27-4691-s001.pdf]

Chemistry–A European Journal

Supporting Information

Tetryl-Tetrylene Addition to Phenylacetylene

Jakob-Jonathan Maudrich,[a] Fatima Diab,[a] Sebastian Weiß,[a] Magda Zweigart,[a]
Klaus Eichele,[a] Hartmut Schubert,[a] Robert Müller,[b] Martin Kaupp,*[b] and
Lars Wesemann*[a]

Supporting Information

Content

1 Crystal structure analysis 2
1.1 Space filling drawing of compound **3** 4
2 NMR data 5
2.1 NMR spectra of compound **4** 6
2.2 NMR spectra of compound **5** 10
2.3 NMR spectra of compound **6** 13
2.4 NMR spectra of compound **7** 15
2.5 NMR spectra of compounds E-**8**, Z-**8** 18
2.6 NMR spectra of compound **9** 25
3 Computational details: structure optimizations, NBO analyses, NMR chemical shift calculations 28

1 Crystal structure analysis

X-ray data were collected with a Bruker Smart APEX II diffractometer with graphite-monochromated Mo Kα radiation or a Bruker APEX II Duo diffractometer with a Mo IμS microfocus tube. The programs used were Bruker's APEX2 v2011.8-0, including SAINT for data reduction, SADABS for absorption correction and SHELXS for structure solution, as well as the WinGX suite of programs version 1.70.01 or the GUI ShelXle, including SHELXL for structure refinement.[1]

Table S1. Crystal structure refinement table of compounds **4**,**5**,**7**-**9**

**compound**	**4**	**5**	**7**	Z-**8**	**9**
Empirical formula	$C_{68}H_{82}GeSn$	$C_{68}H_{82}GePb$	$C_{64}H_{60}Pb_2$	$C_{68}H_{82}PbSn$	$C_{84}H_{81}AlF_{36}GeO_4Sn$
M_r [g mol^{-1}]	1090.61	4716.44	1243.52	1225.21	2056.74
T [K]	100(2)	100(2)	100(2)	100(2)	100(2)
λ [Å]	0.71073	0.71073	0.71073	0.71073	0.71073
Crystal system	orthorhombic	orthorhombic	Monoclinic	orthorhombic	monoclinic
Space group	Pn	$Pna2_1$	$P2_1/n$	Pn	$P2_1/c$
Z	4	4	2	4	4
a [Å]	23.8885(4)	24.013(5)	11.32002(2)	23.7423(9)	13.9823(3)
b [Å]	12.4057(2)	12.366(3)	22.1569(5)	12.2101(5)	18.3463(4)
c [Å]	19.4664(3)	19.715(4)	11.5363(2)	20.0880(8)	34.5826(7)
α [°]	90	90	90	90	90
β [°]	90	90	116.5680	90	95.2130(10)
γ [°]	90	90	90	90	90
V [Å^3]	5768.94(16)	5854(2)	2587.99(9)	5823.4(4)	8834.6(3)
D_{calc} [g cm^{-3}]	1.256	1.338	1.596	1.397	1.546
μ [mm^{-1}]	0.993	3.424	6.536	3.355	0.757
F(000)	2288	2416	1216	2488	4144
Crystal size [mm]	0.17×0.13×0.11	0.18x0.16x0.14	0.14x0.12x0.10	0.16×0.14×0.12	0.16×0.15×0.14
θ range[°]	1.705-27.118	3.394-28.324	3.391-27.830	1.715-26.454	1.622-28.347
Limiting indices	$-30 \le h \le 30$	$-31 \le h \le 0$	$-14 \le h \le 13$	$-28 \le h \le 29$	$-18 \le h \le 15$
	$-15 \le k \le 15$	$-16 \le k \le 16$	$-29 \le k \le 27$	$-15 \le k \le 12$	$-24 \le k \le 24$
	$-24 \le l \le 22$	$-26 \le l \le 26$	$-15 \le l \le 14$	-24≤l≤25	$-46 \le l \le 45$
Collected refl.	79534	14258	6114	40437	152185
Independent refl.	12173	11311	5116	11447	22020
R_{int}	0.0367	0.0445	0.0385	0.0542	0.0440
completeness	0.998	0.98	0.995	0.991	0.997
Absorption corr.	multi-scan	multi-scan	multi-scan	multi-scan	multi-scan
Trans. (max., min.)	0.7455, 0.6879	0.7457, 0.6084	0.7456, 0.5926	0.7454, 0.6004	0.7457, 0.6949
Parameter/restraints	645/1	651/2	317/72	652/2	1428/0
R_1, ωR_2 [$I > 2\sigma(I)$]	0.0300, 0.0623	0.0427, 0.0972	0.0397, 0.0621	0.0342, 0.0601	0.0428, 0.0948
R_1, ωR_2 (all data)	0.0363, 0.0648	0.0624, 0.1020	0.0541, 0.0649	0.0526, 0.0650	0.0641, 0.1028
GooF on F^2	1.021	1.055	1.172	0.931	1.025
$\Delta\rho_{max,min}$ [e·Å^{-3}]	0.940, -0.418	2.750, -0.949	2.691, -2.152	0.926, -1.152	1.412, -0.735
CCDC	2046116	2046112	2046113	2046115	2046114

1.1 Space filling drawing of compound **3**

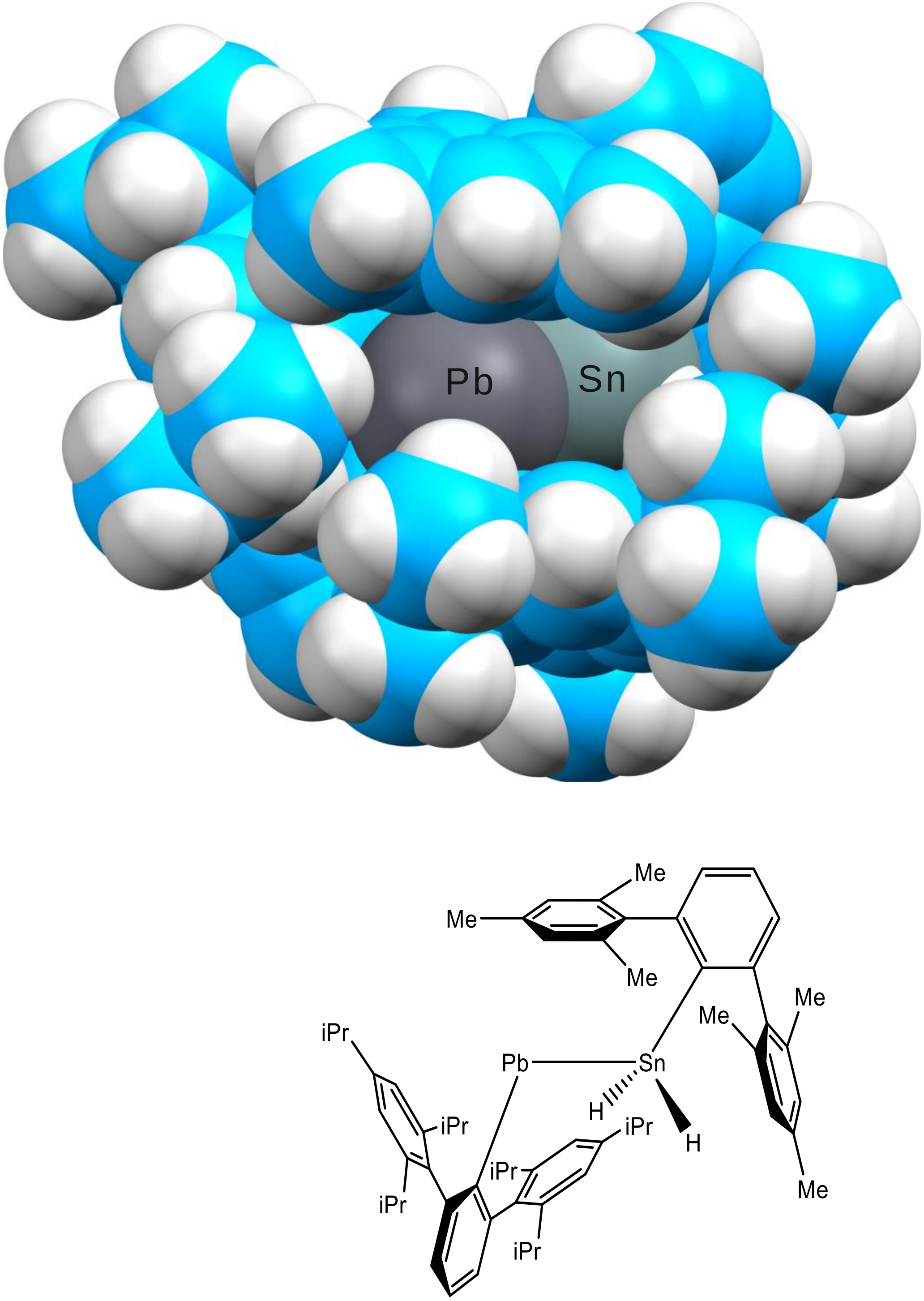

Figure S1. Space filling drawing of **3**.

2 NMR data

NMR spectra were recorded on a Bruker DRX-250 NMR spectrometer (^{1}H, 250.13 MHz; ^{13}C, 62.90 MHz; ^{119}Sn, 93.28 MHz, ^{207}Pb, 52.29 Hz) equipped with a 5 mm ATM probe head, a Bruker AvanceII+400 NMR spectrometer (^{1}H, 400.11 MHz; ^{13}C, 100.61 MHz) equipped with a 5 mm QNP (quad nucleus probe) head and a Bruker AvanceII+500 NMR-spectrometer (^{1}H, 500.13 MHz; ^{13}C, 125.76 MHz; ^{119}Sn, 186.50 MHz) equipped with a 5 mm ATM probe head and a setup for variable temperature. The chemical shifts are reported in δ values in ppm relative to external $SiMe_4$ (^{1}H, ^{13}C), $SnMe_4$ (^{119}Sn) or $PbMe_4$ (^{207}Pb) using the chemical shift of the solvent ^{2}H resonance frequency and Ξ = 25.145020 % for ^{13}C, Ξ = 37.290632 % for ^{119}Sn and Ξ = 20.920599% for ^{207}Pb.[2] The multiplicity of the signals is abbreviated as s = singlet, d = doublet, t = triplet, quint = quintet, sept = septet and m = multiplet or unresolved. The proton and carbon signals were assigned by detailed analysis of ^{1}H, $^{13}C\{^{1}H\}$, ^{1}H-^{1}H COSY, ^{1}H-^{13}C HSQC, ^{1}H-^{13}C HMBC and $^{13}C\{^{1}H\}$ DEPT 135 spectra.

2.1 NMR spectra of compound **4**

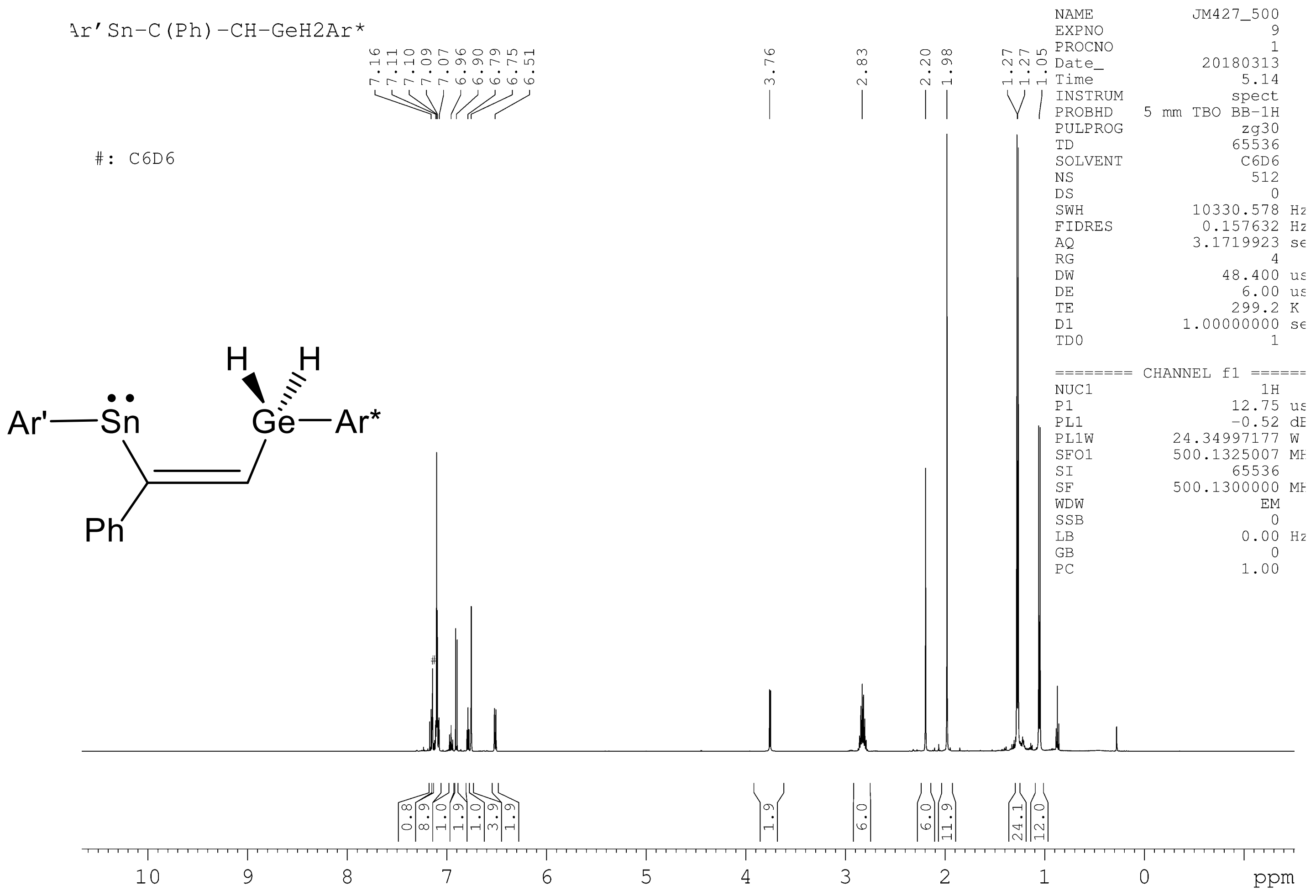

Figure S2. ^{1}H NMR of compound **4**.

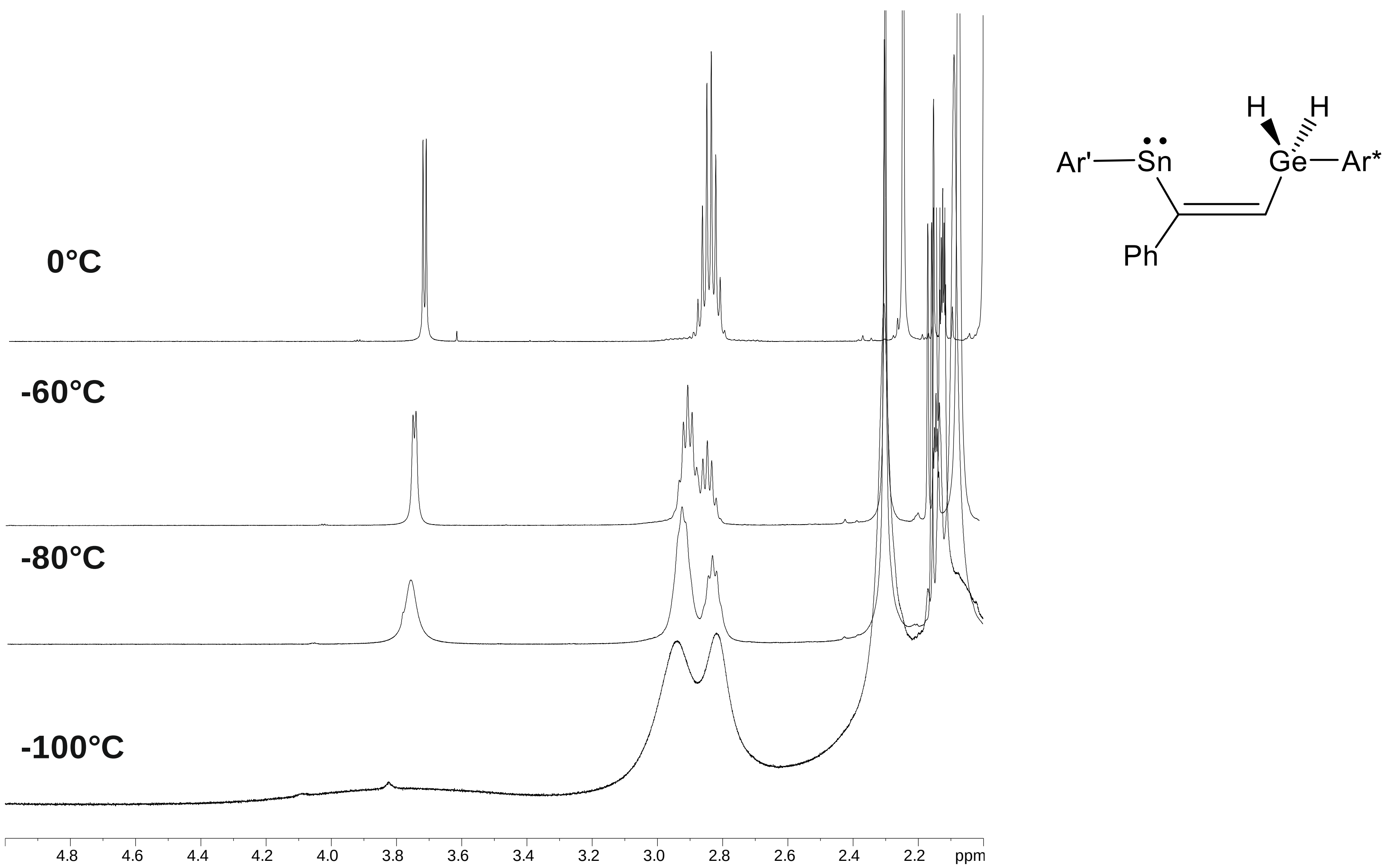

Figure S3. ^{1}H NMR of compound **4** at different temperatures.

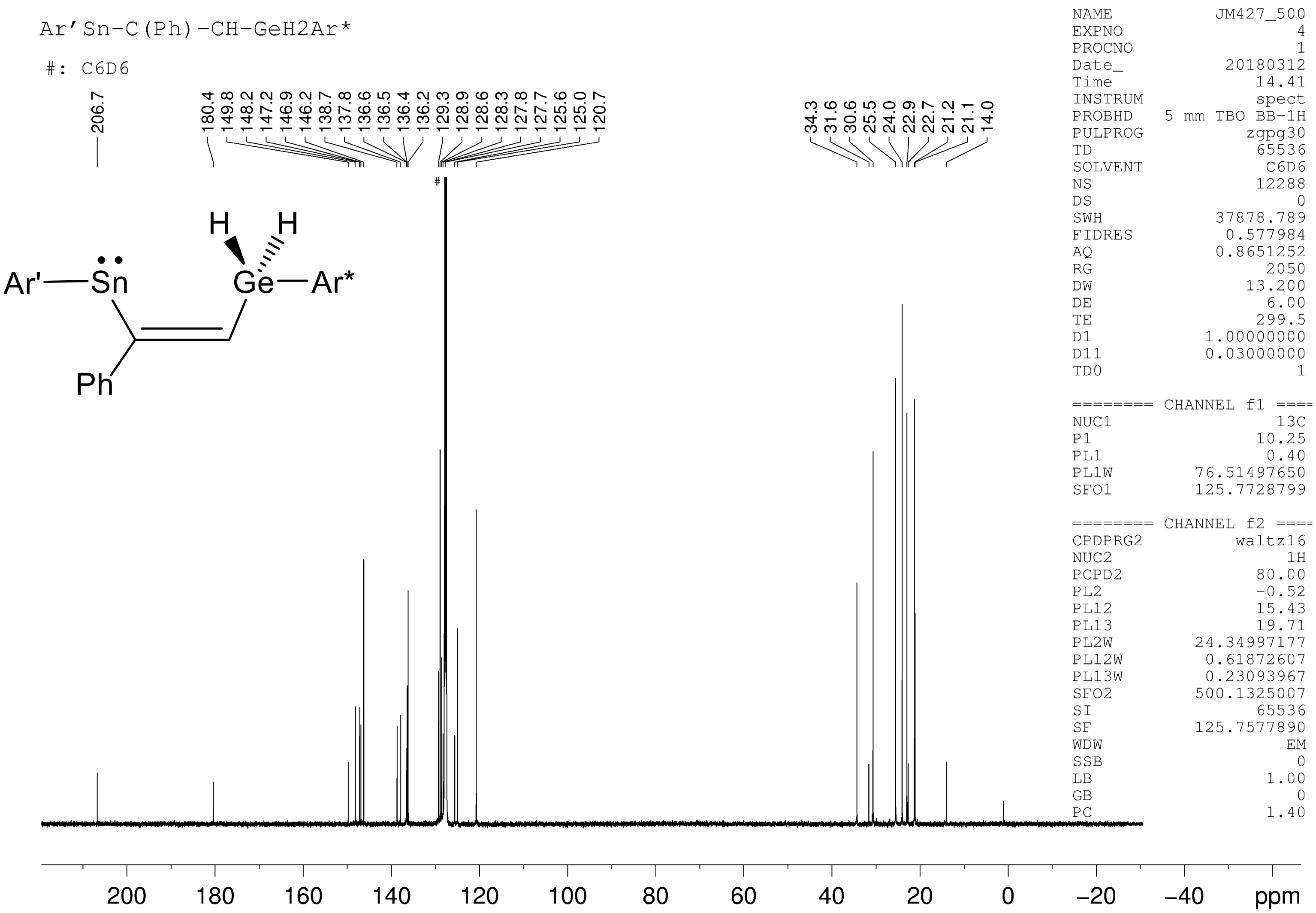

Figure S4. $^{13}C\{^{1}H\}$ NMR spectrum of compound **4**.

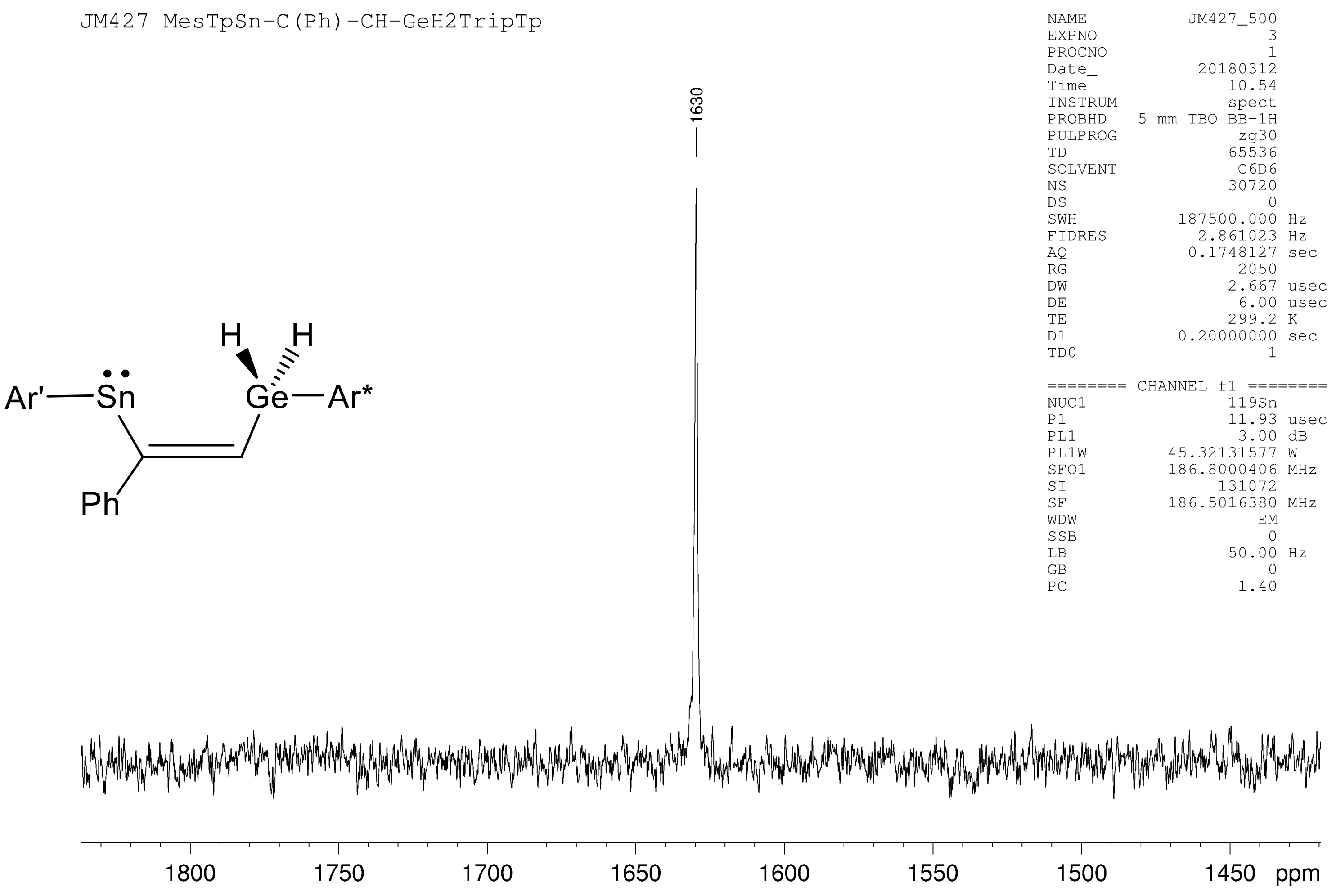

Figure S5. ^{119}Sn NMR spectrum of compound **4**.

2.2 NMR spectra of compound **5**

Ar*GeH$_2$C(H)C(Ph)PbAr‘ in C_6D_6

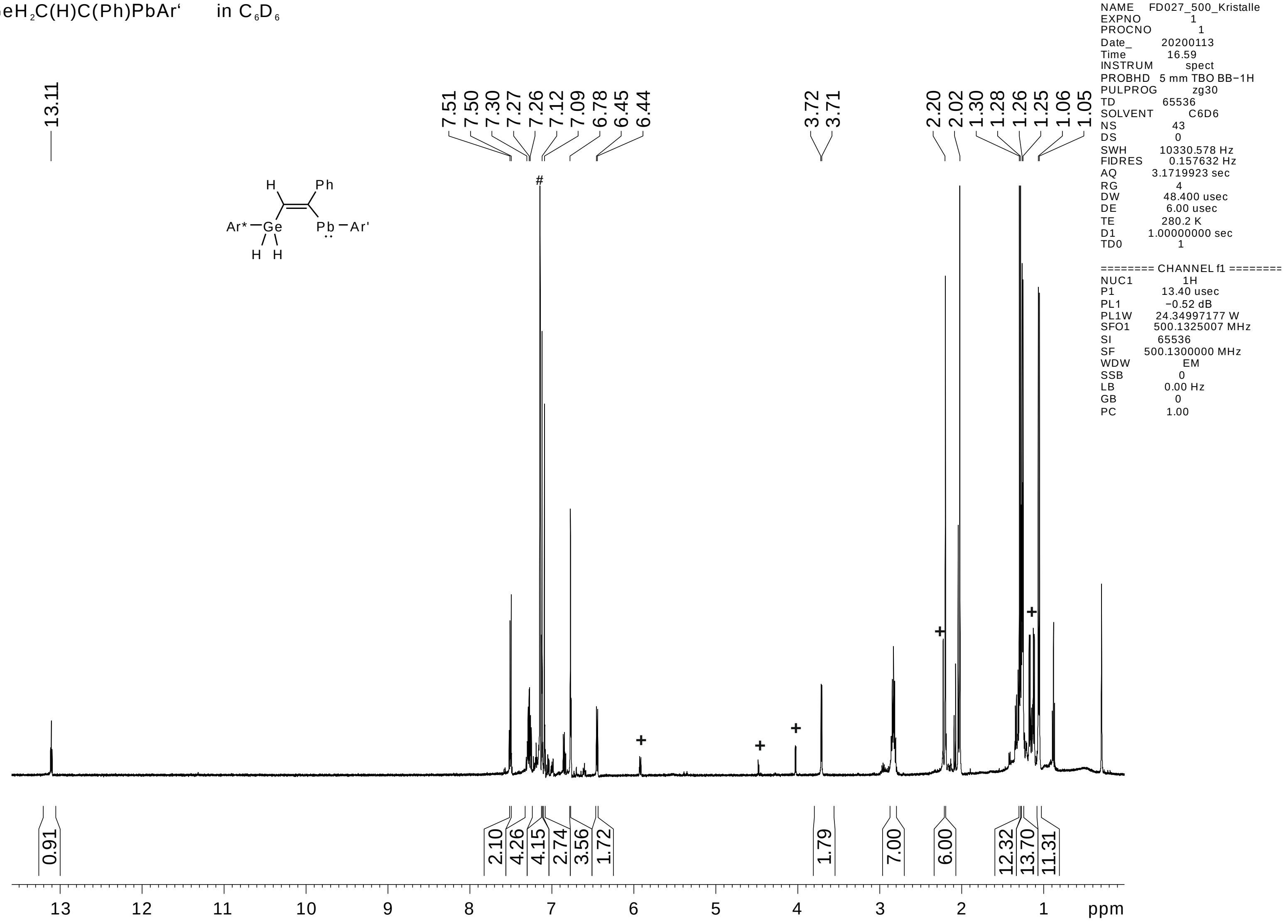

Figure S6. ^{1}H NMR of compound **5**. (# = solvent, + = undefined impurities)

Ar*GeH$_2$C(H)C(Ph)PbAr' in C_6D_6

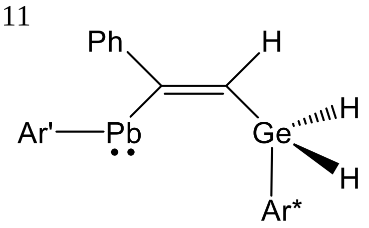

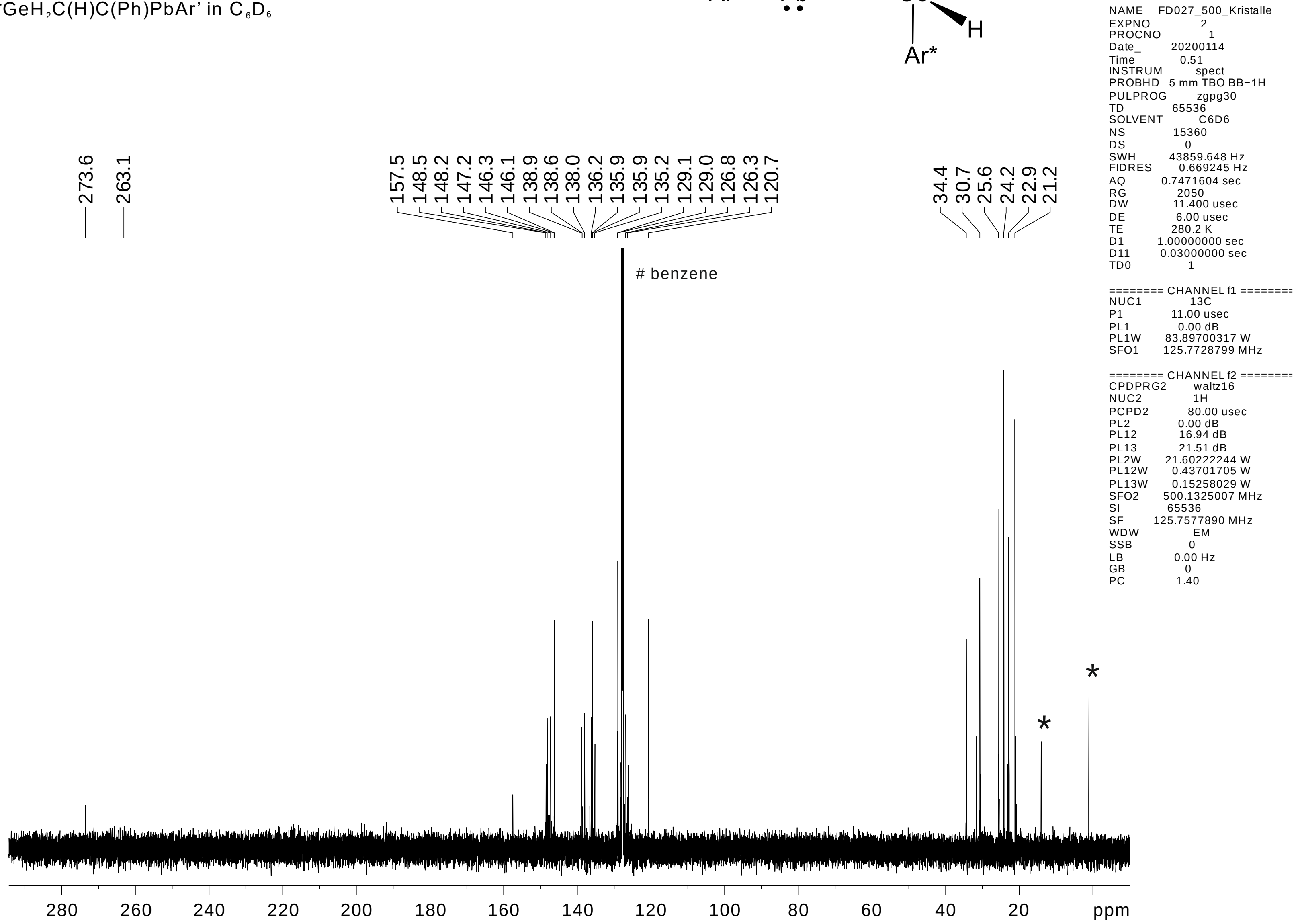

Figure S7. $^{13}C\{^1H\}$ NMR spectrum of compound **5**. (* = impurities)

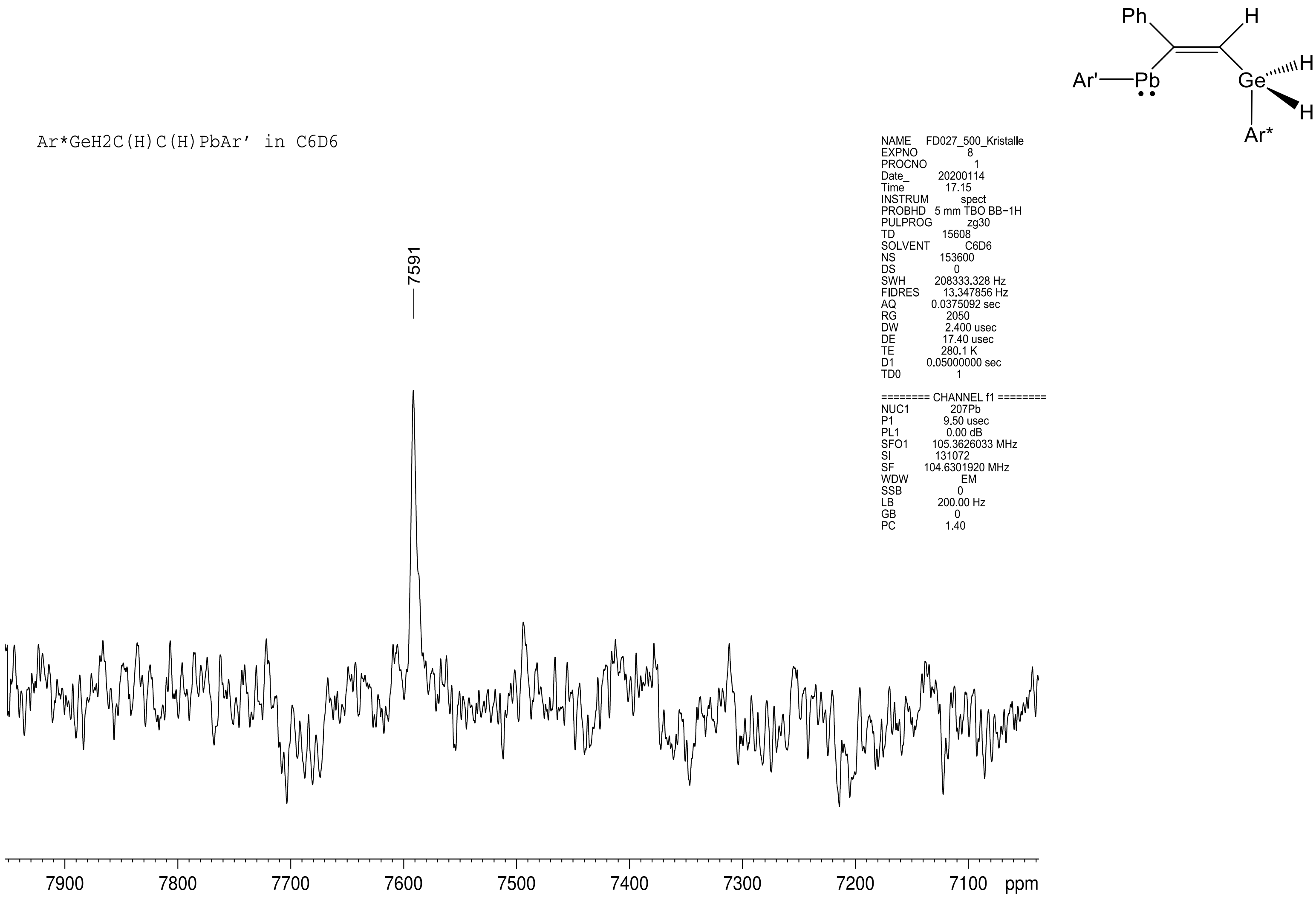

Figure S8. ^{207}Pb NMR spectrum of compound **5**.

2.3 NMR spectra of compound **6**

Ar*GeH$_2$C(H)C(H)(Ph) (**6**) in C_6D_6

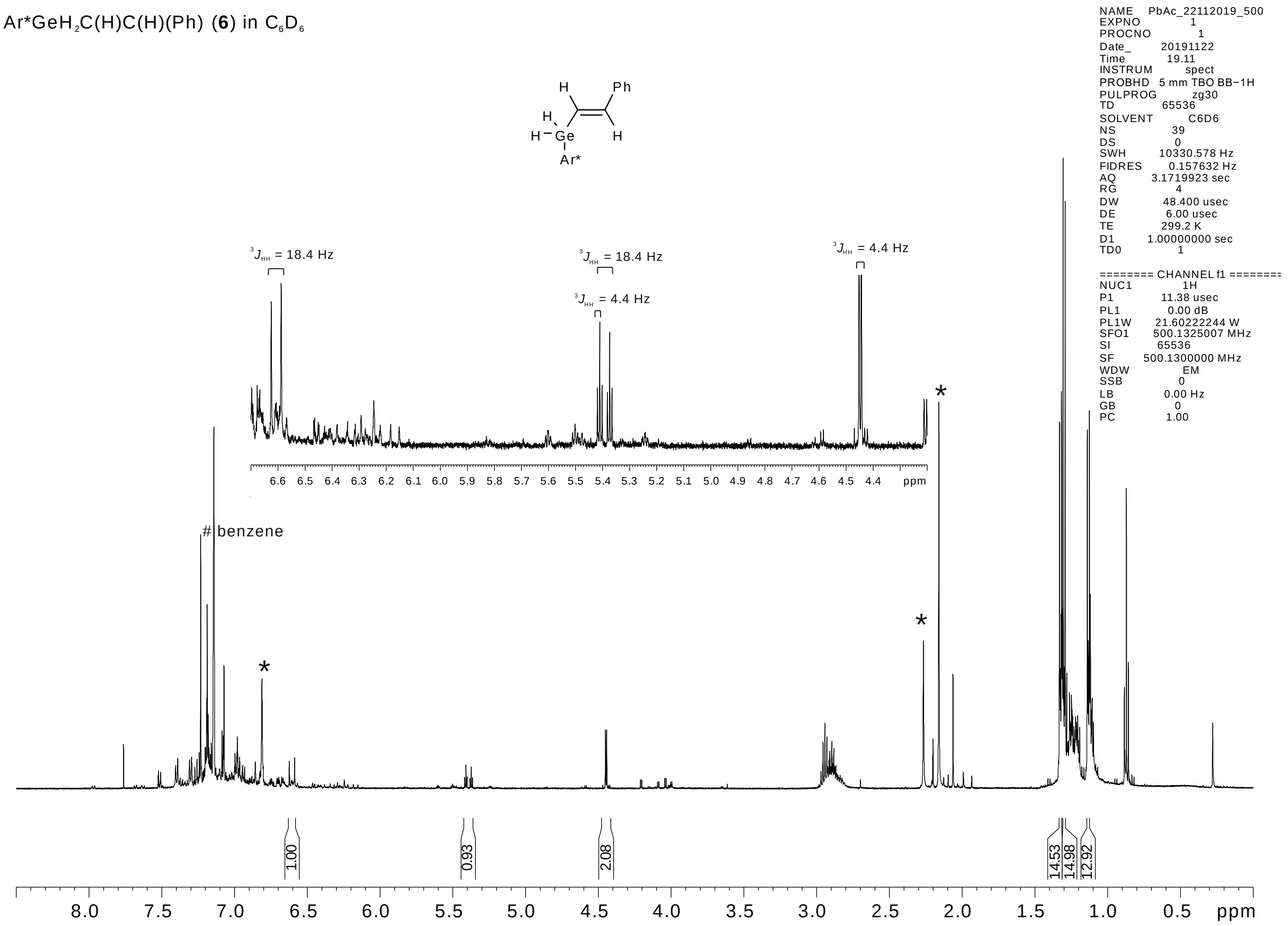

Figure S9. ^{1}H NMR of compound **6**. (* = **7** as impurity)

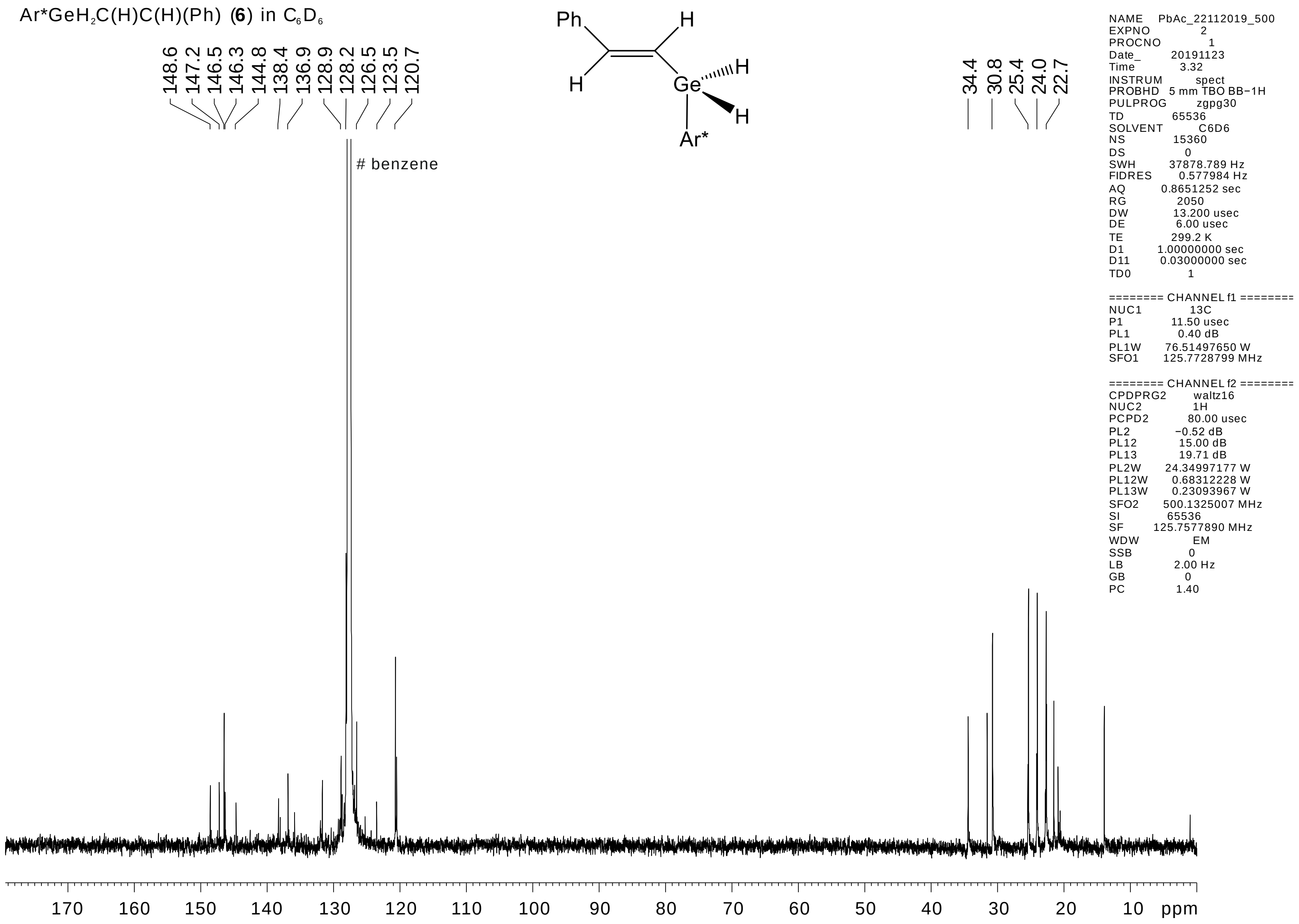

Figure S10. $^{13}C\{^1H\}$ NMR spectrum of compound **6**.

2.4 NMR spectra of compound **7**

$(Ar'PbCCPh)_2$ in C_6D_6

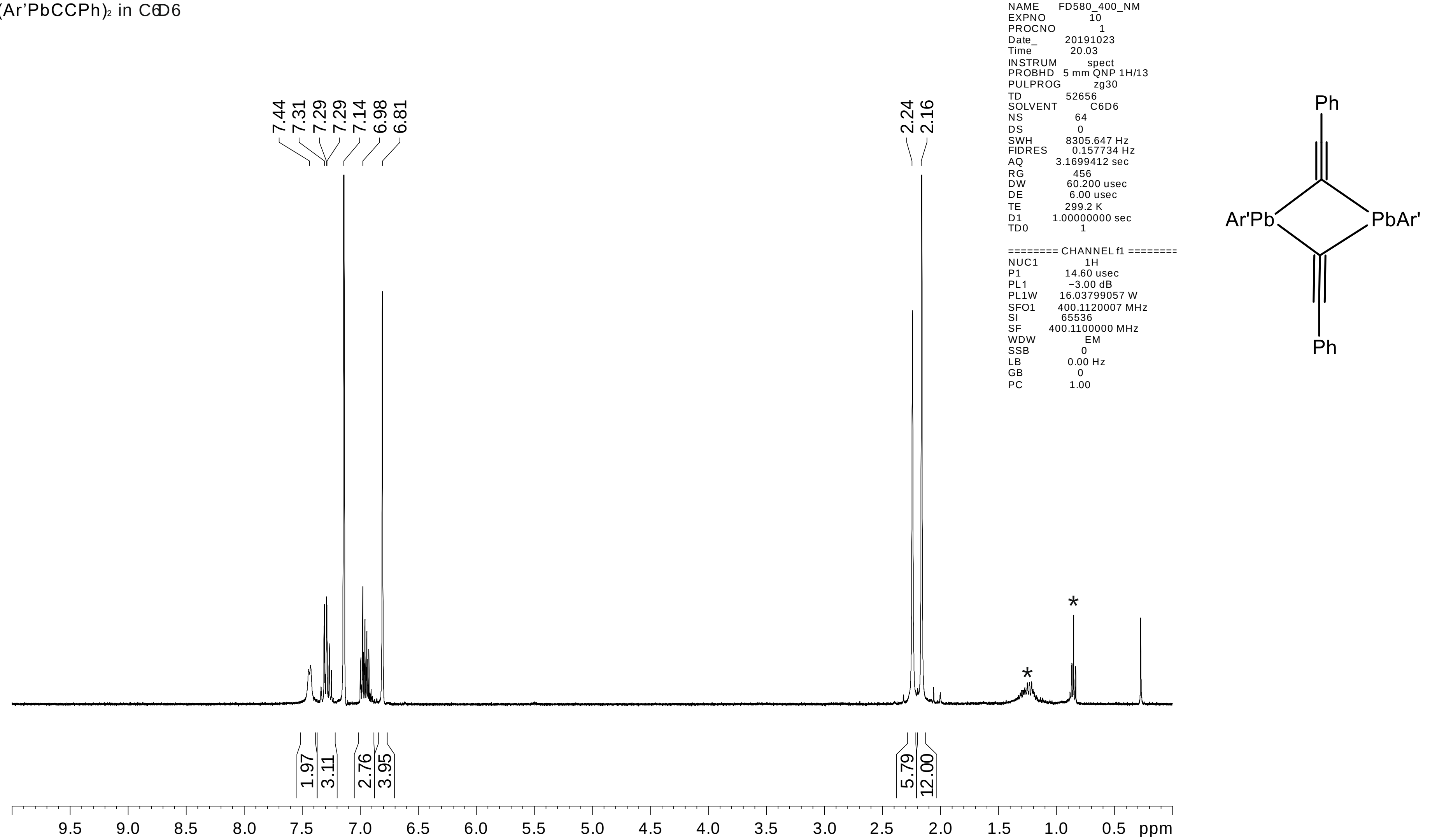

Figure S11. ^{1}H NMR spectrum of compound **7**. (* = hexane solvent)

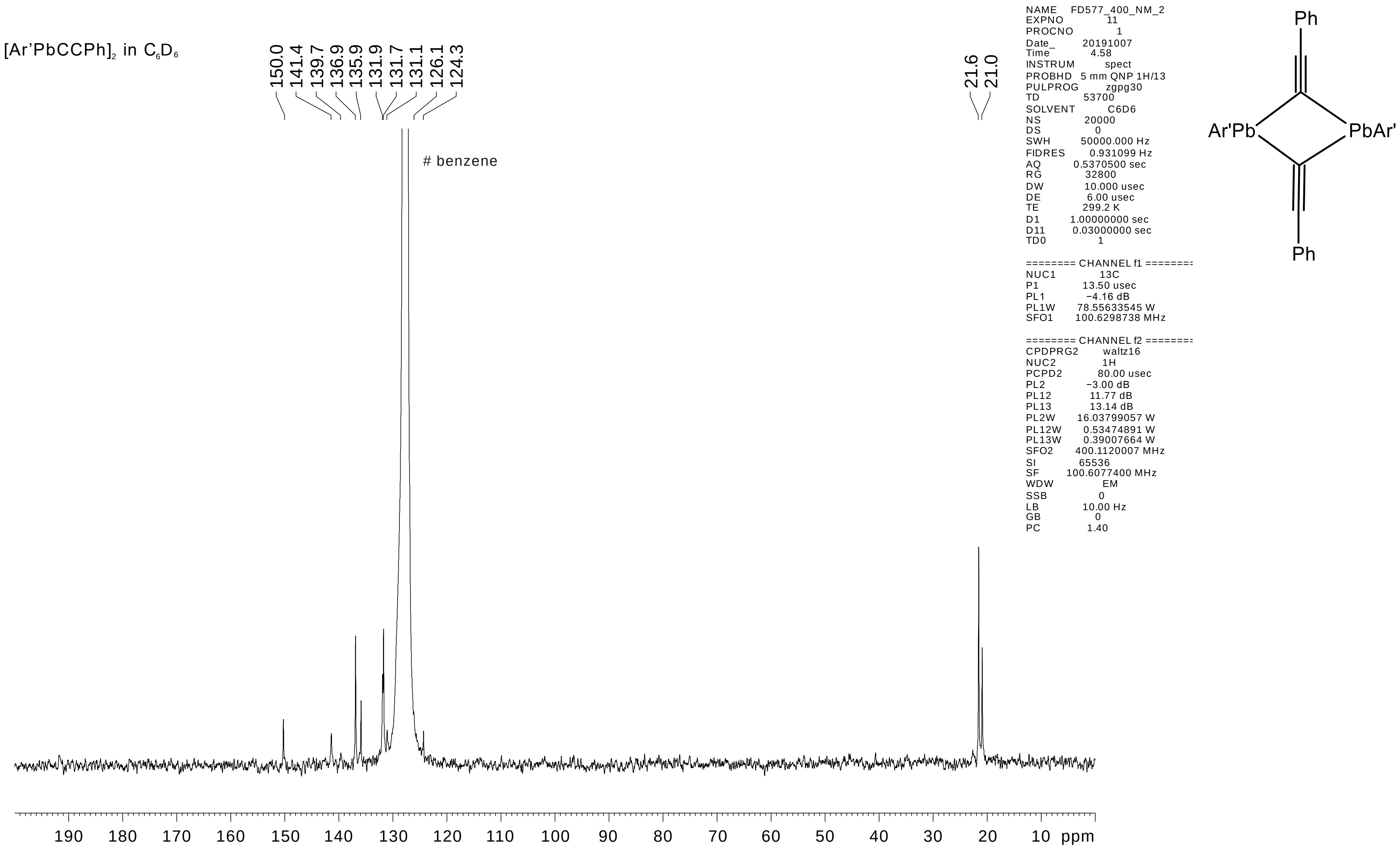

Figure S12. $^{13}C\{^1H\}$ NMR spectrum of compound **7**.

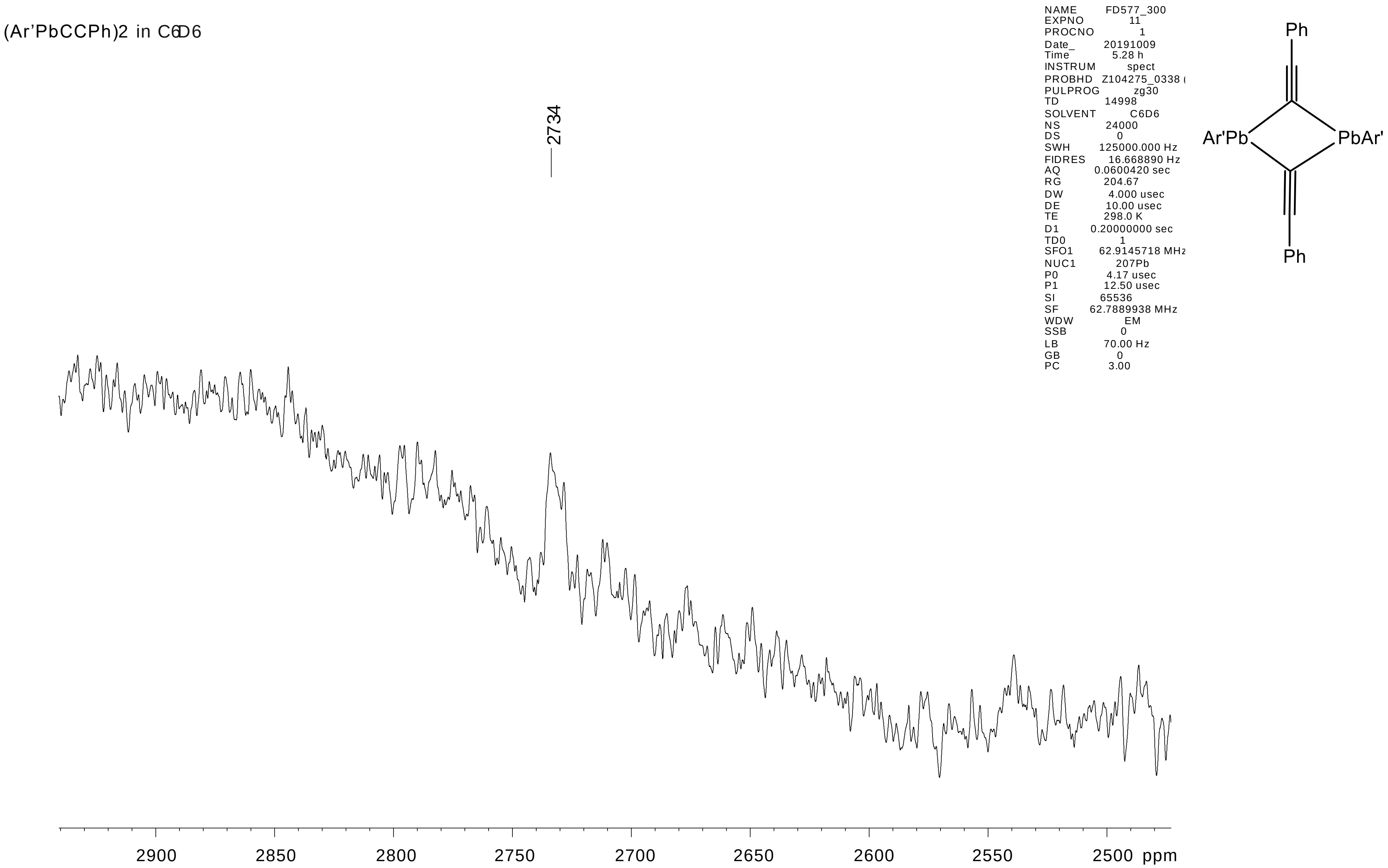

Figure S13. ^{207}Pb NMR spectrum of compound **7**.

2.5 NMR spectra of compounds E-**8**, Z-**8**

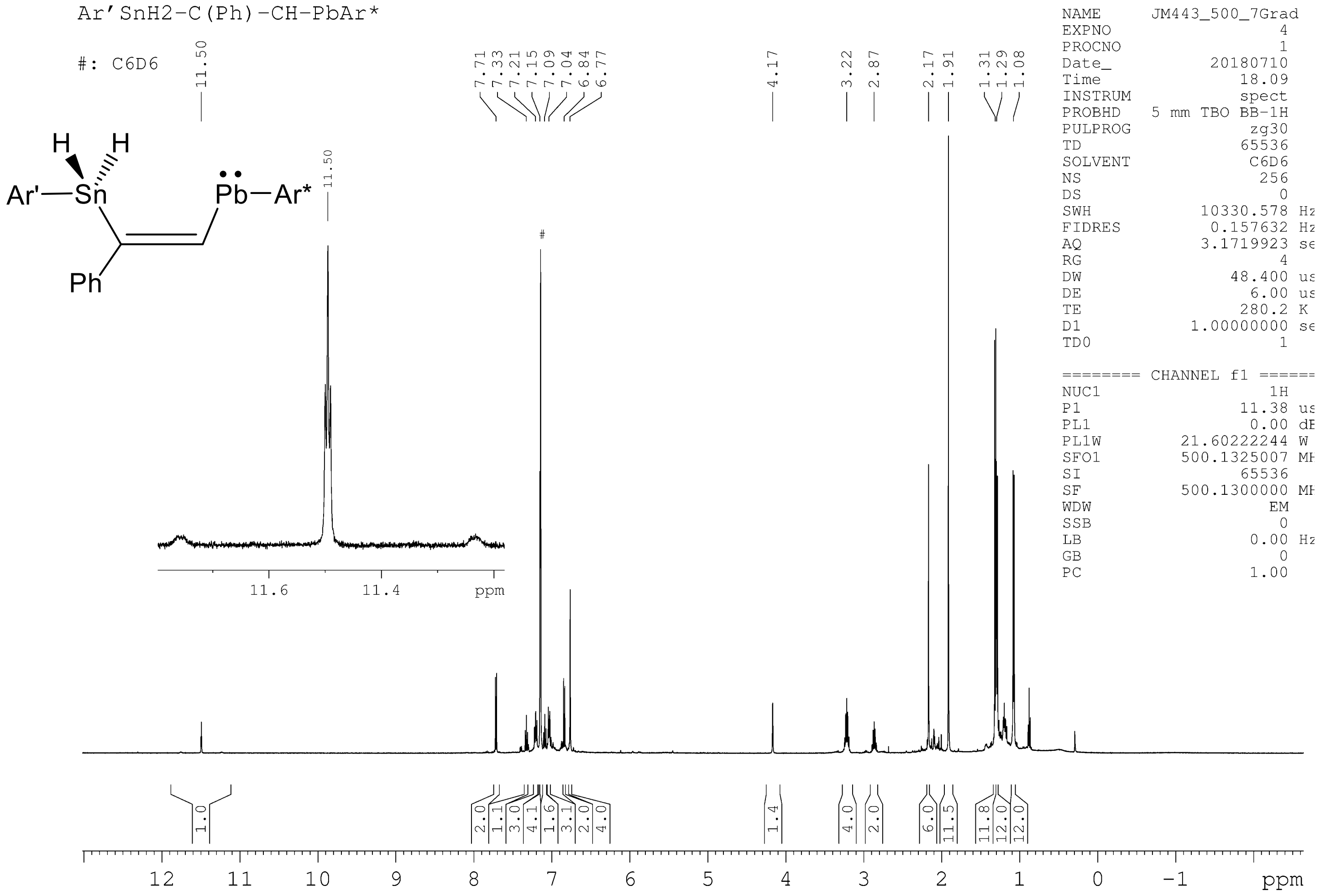

Figure S14. ^{1}H NMR of compound Z-**8**.

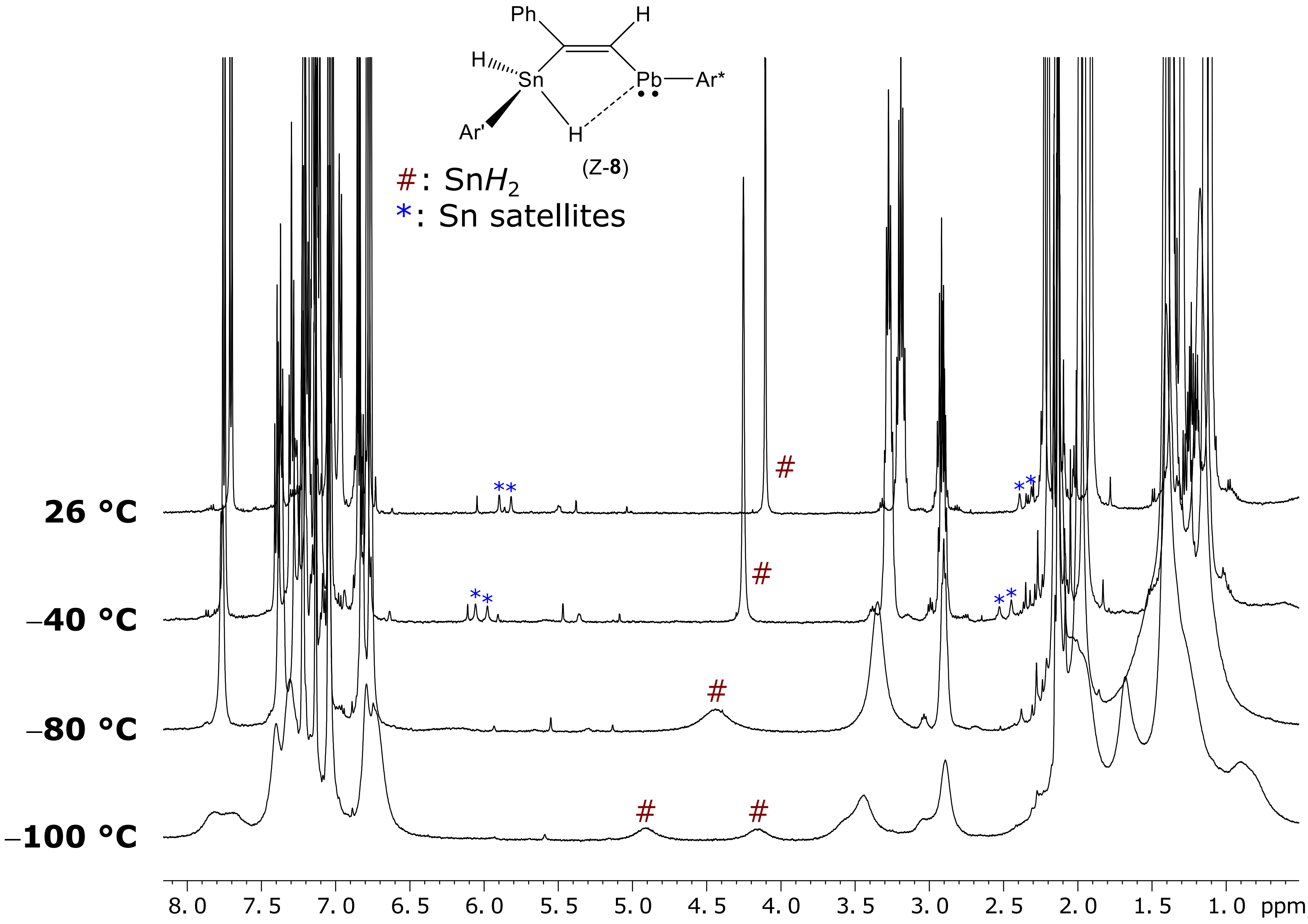

Figure S15. ^{1}H NMR of compound Z-**8** at different temperatures, showing at -100°C two signals for the SnH_2 moiety due to interaction with lead.

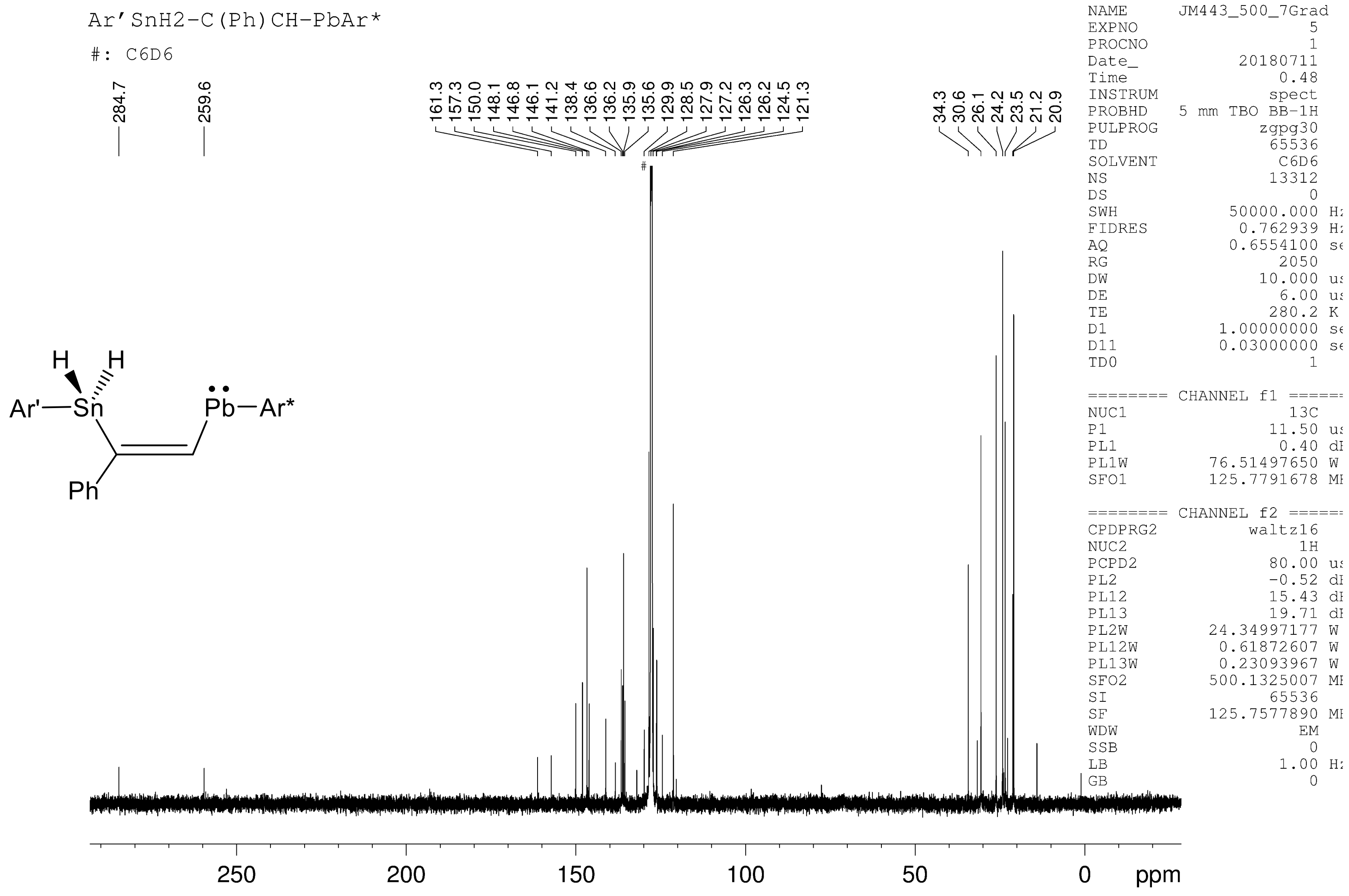

Figure S16. $^{13}C\{^{1}H\}$ NMR spectrum of compound Z-**8**.

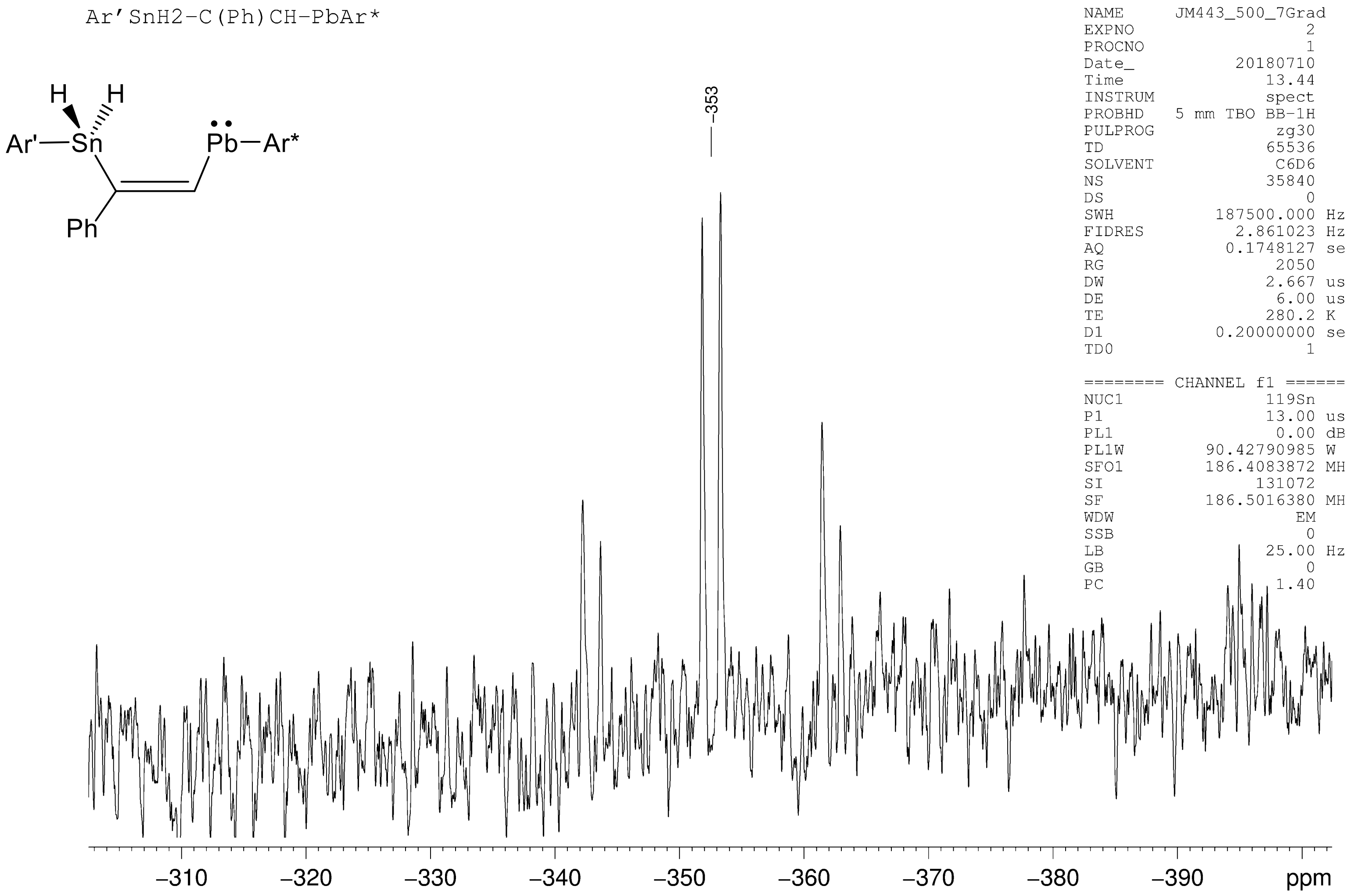

Figure S17. ^{119}Sn NMR spectrum of compound Z-**8**.

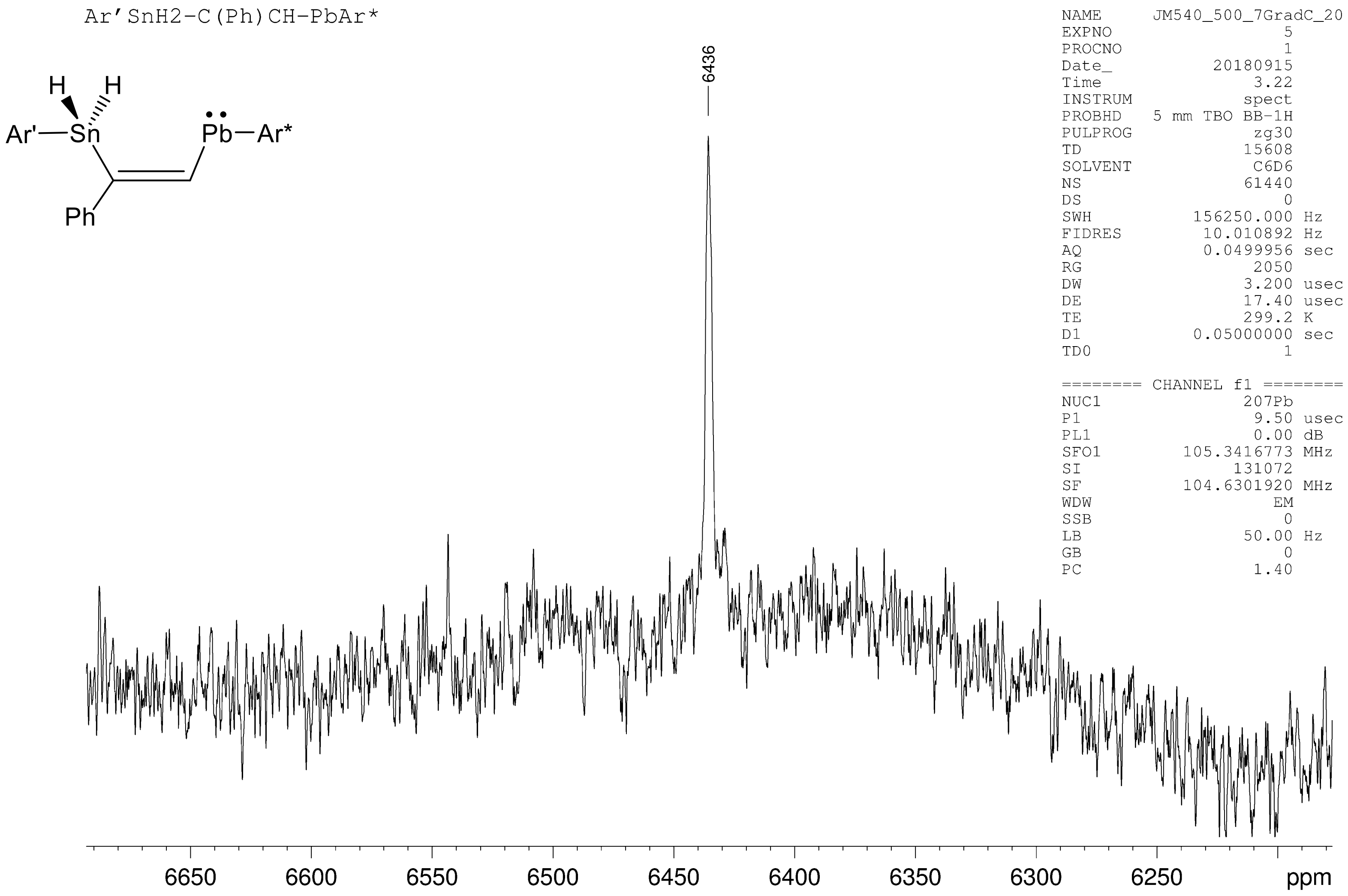

Figure S18. ^{207}Pb NMR spectrum of compound Z-**8**.

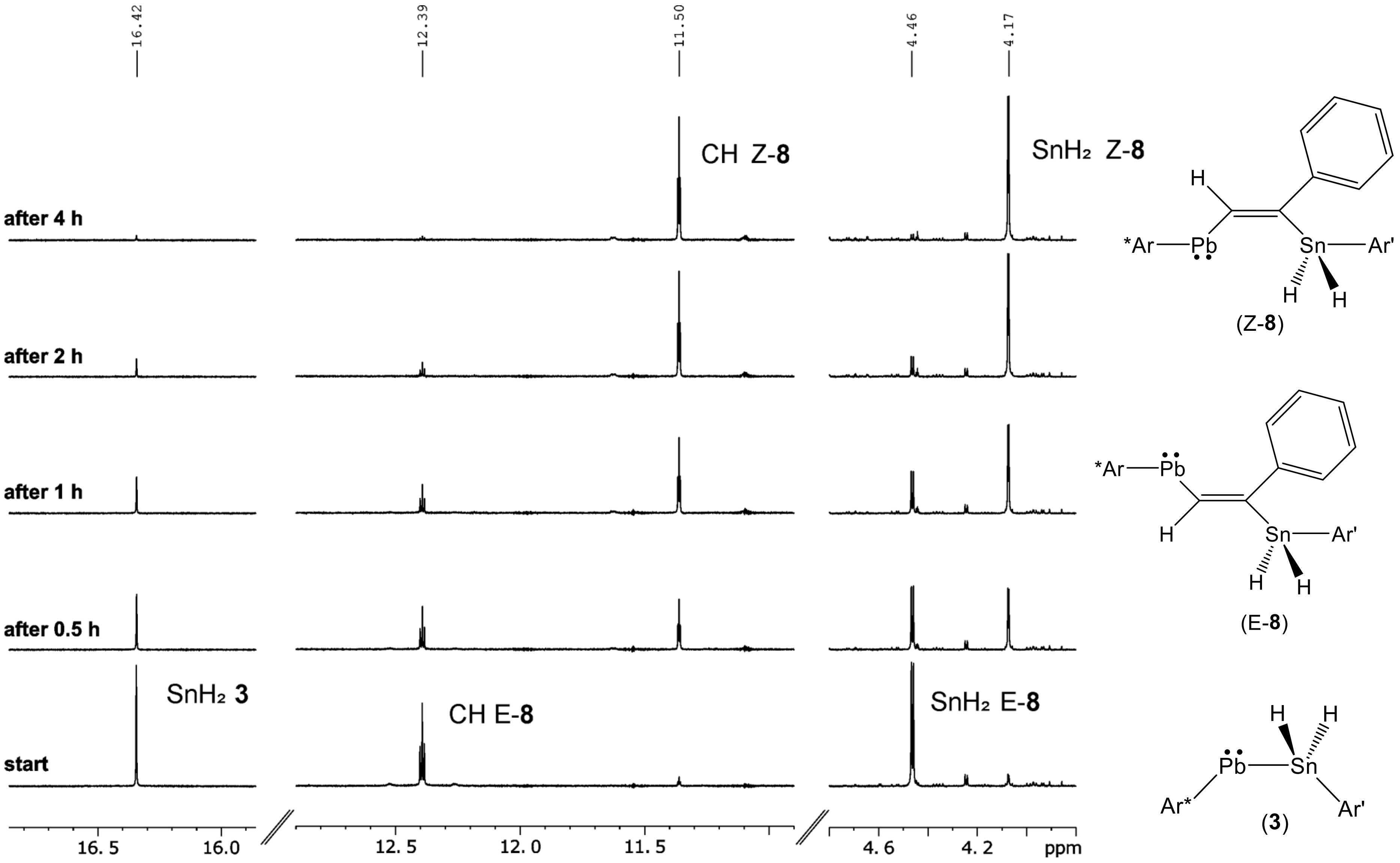

Figure S19. ^{1}H NMR reaction of **3** with excess PhCCH. Start: fast formation of isomer E-**8** due to high excess of PhC≡CH and formation of Z-**8**.

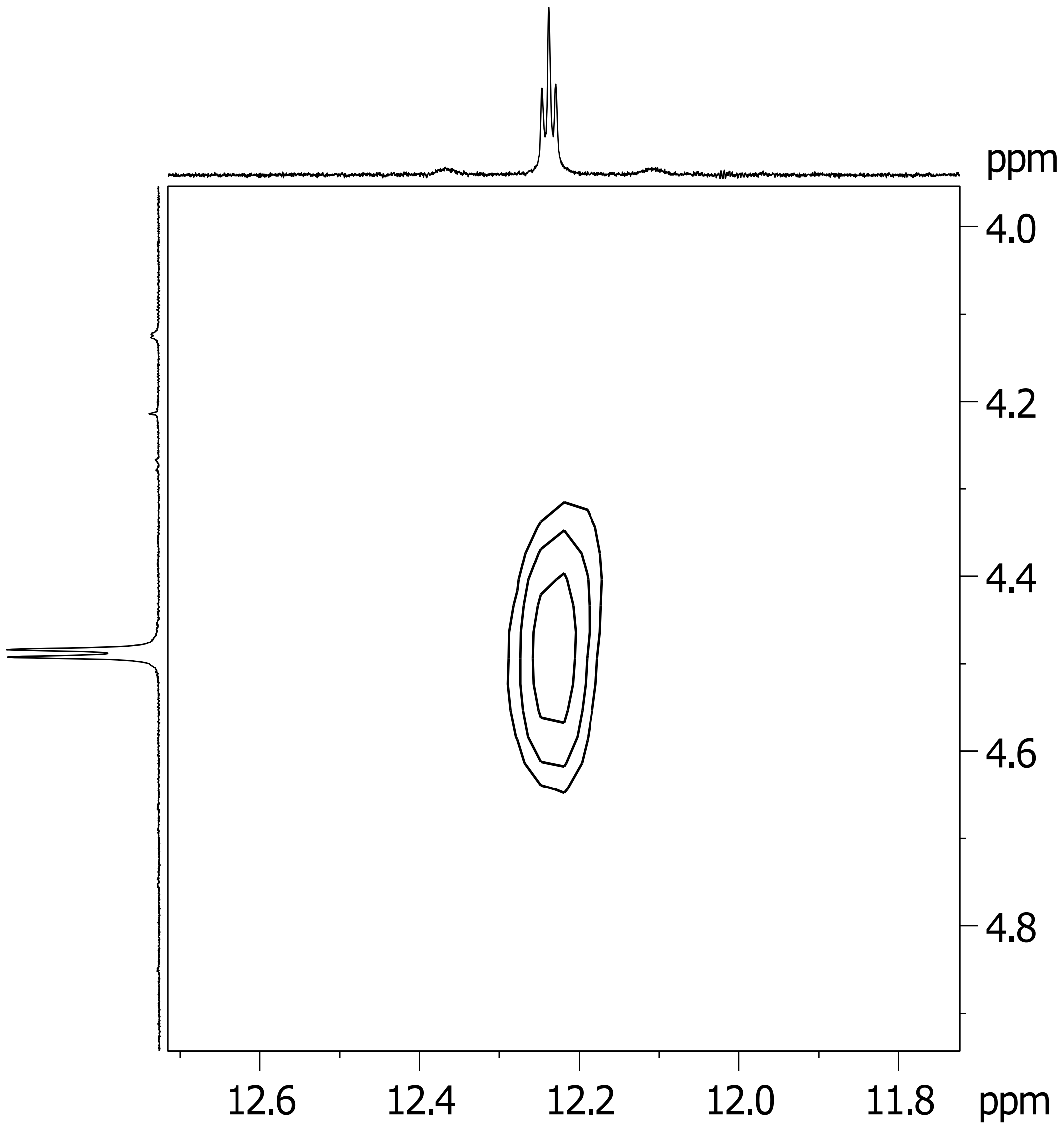

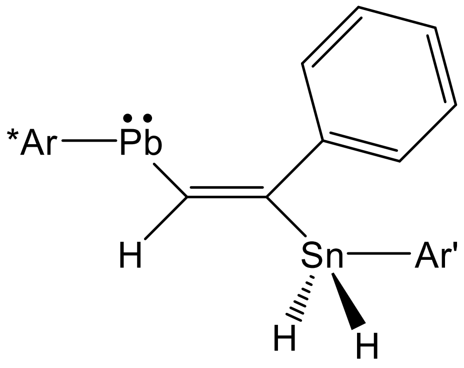

(E-**8**)

Figure S20. NOESY (0°C) of isomer E-**8** showing a cross peak between the cis orientated CH and SnH_2 units.

2.6 NMR spectra of compound **9**

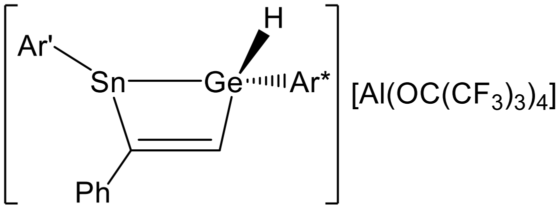

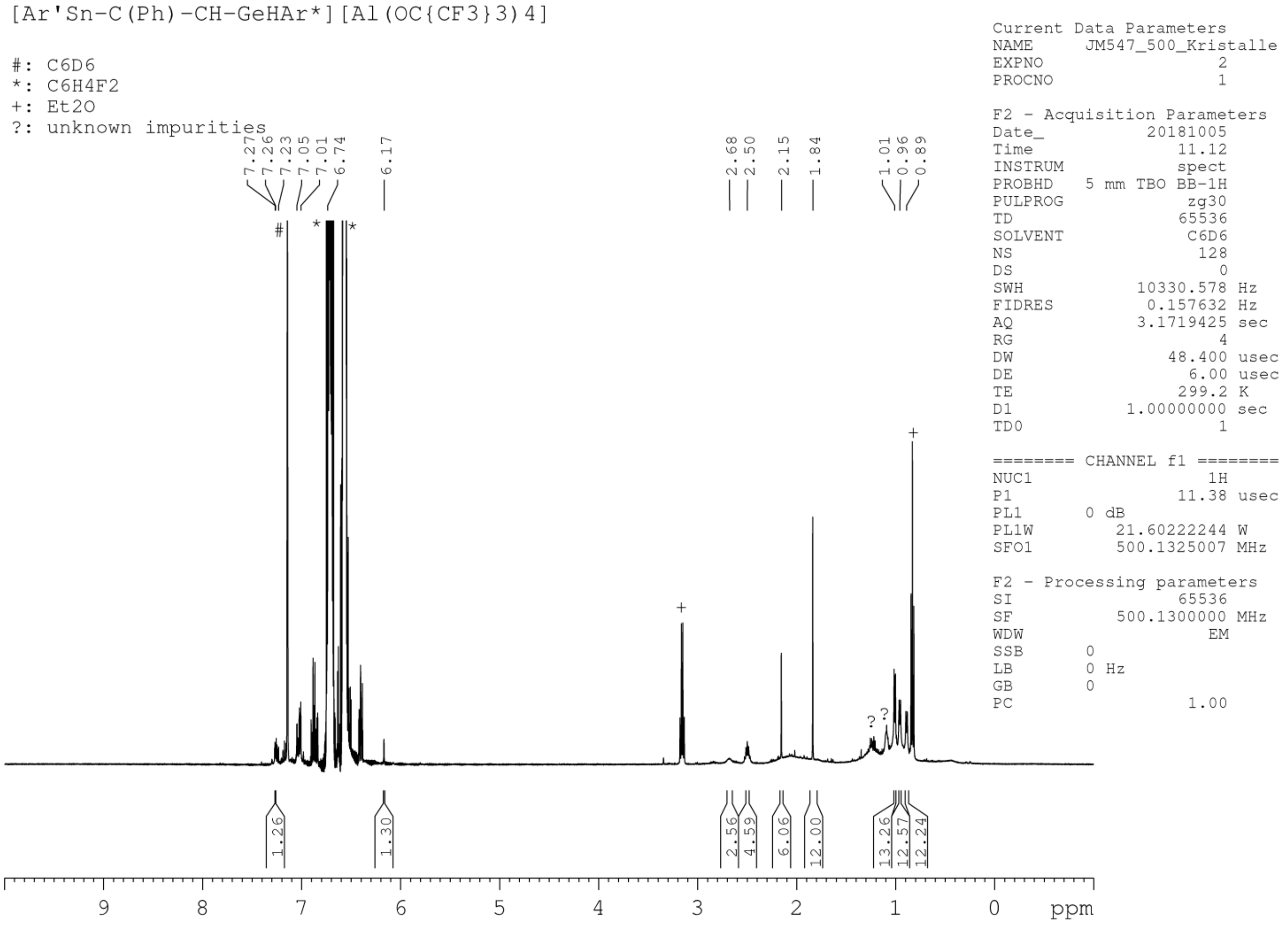

Figure S21. ^{1}H NMR spectrum of **9**.

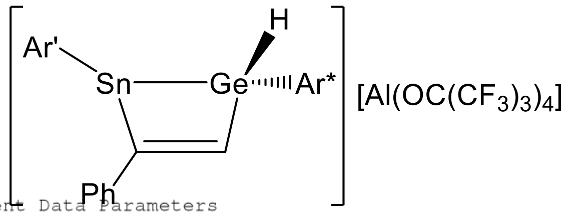

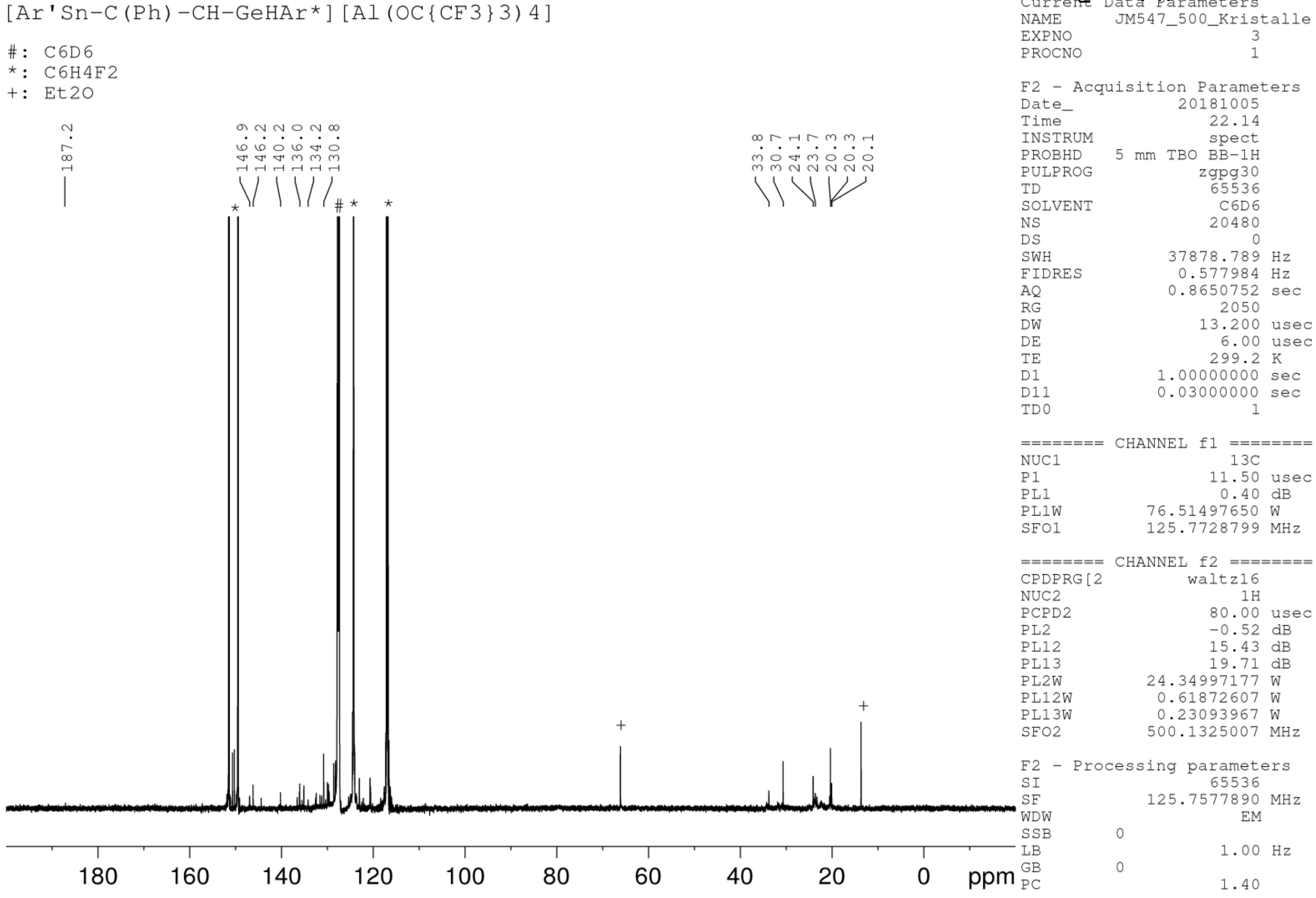

Figure S22. $^{13}C\{^{1}H\}$ NMR spectrum of **9**.

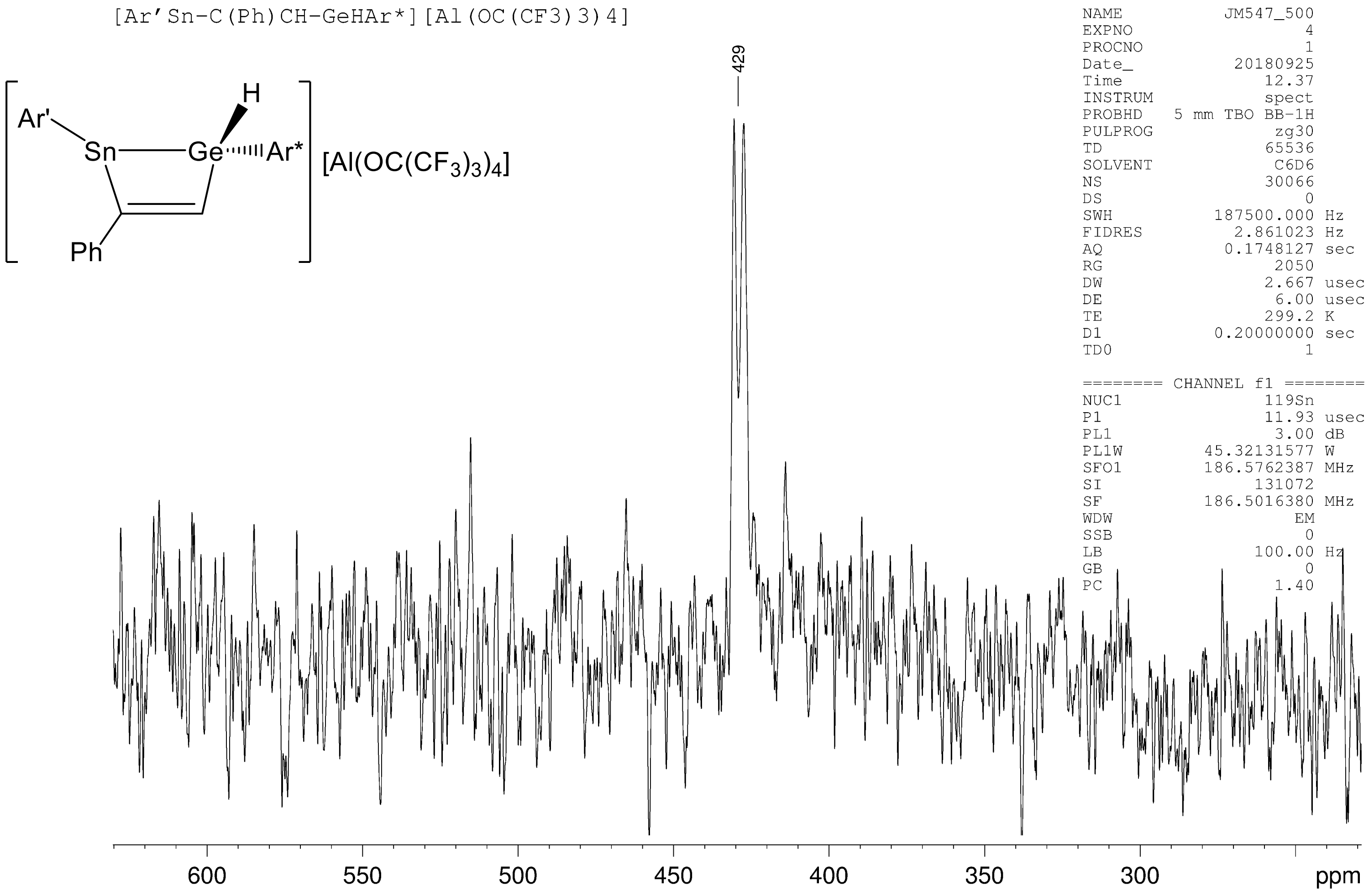

Figure S23. ^{119}Sn NMR spectrum of compound **9**.

3 Computational details: structure optimizations, NBO analyses, NMR chemical shift calculations

Structure optimizations at DFT level were carried out with Gaussian09.[3] On the basis of the molecular structure determined by single crystal X-Ray diffraction, the structures of compounds **5**, Z-**8** and **9** were optimized using the BP86[4] functional and def2-TZVP[5] basis sets for all atoms, except for Sn and Pb.[4-6] For the latter atoms, Stuttgart-Dresden effective core potentials (ECPs) were employed, in combination with the originally associated valence basis sets.[4-6] Dispersion corrections were included by adding Grimme's D3 corrections with Becke-Johnson damping, D3(BJ).[7] In all cases the structures obtained were characterized as minima using harmonic vibrational frequency analyses. Natural bond orbitals were obtained using the NBO 6.0 software.[8] Plots were generated with the software Chemcraft.[9]

On the basis of the optimized structures of **5**, Z-**8** and **9** NMR chemical shifts were computed at SO-ZORA level with ADF,[10] including the crucial exchange correlation kernel (PBE density functional, basis sets: TZ2P Sn, C, TZP H).[11] The obtained ^{119}Sn, ^{13}C and ^{1}H NMR shieldings were converted to chemical shifts (δ, in ppm) relative to the shieldings of tetramethyltin ($\sigma[^{119}Sn]=2901.613$) and tetramethylsilane (TMS; $\sigma[^{13}C]=188.25$; $\sigma[^{1}H]=31.84$), calculated at identical computational level. [11] Nuclear shieldings of Z-**8** were analyzed using the NBO 6.0 software.[8b, 12] Additional GIAO-DFT shielding calculations for Z-**8** used the four-component (4c) relativistic matrix Dirac-Kohn-Sham (mDKS) method implemented in the Respect program, version 5.1.0,[13] and the PBE density functional, in conjunction with fully uncontracted IGLO-II basis sets for the light atoms (C, H)[14] and Dyall valence triple-ζ basis sets (Dyall-VTZ) for Sn and Pb.[15] The obtained ^{13}C and ^{1}H shieldings were converted to chemical shifts (δ, in ppm) relative to the shieldings of tetramethylsilane (TMS; $\sigma[^{13}C]=182.890$; $\sigma[^{1}H]=31.584$), calculated at identical computational level.

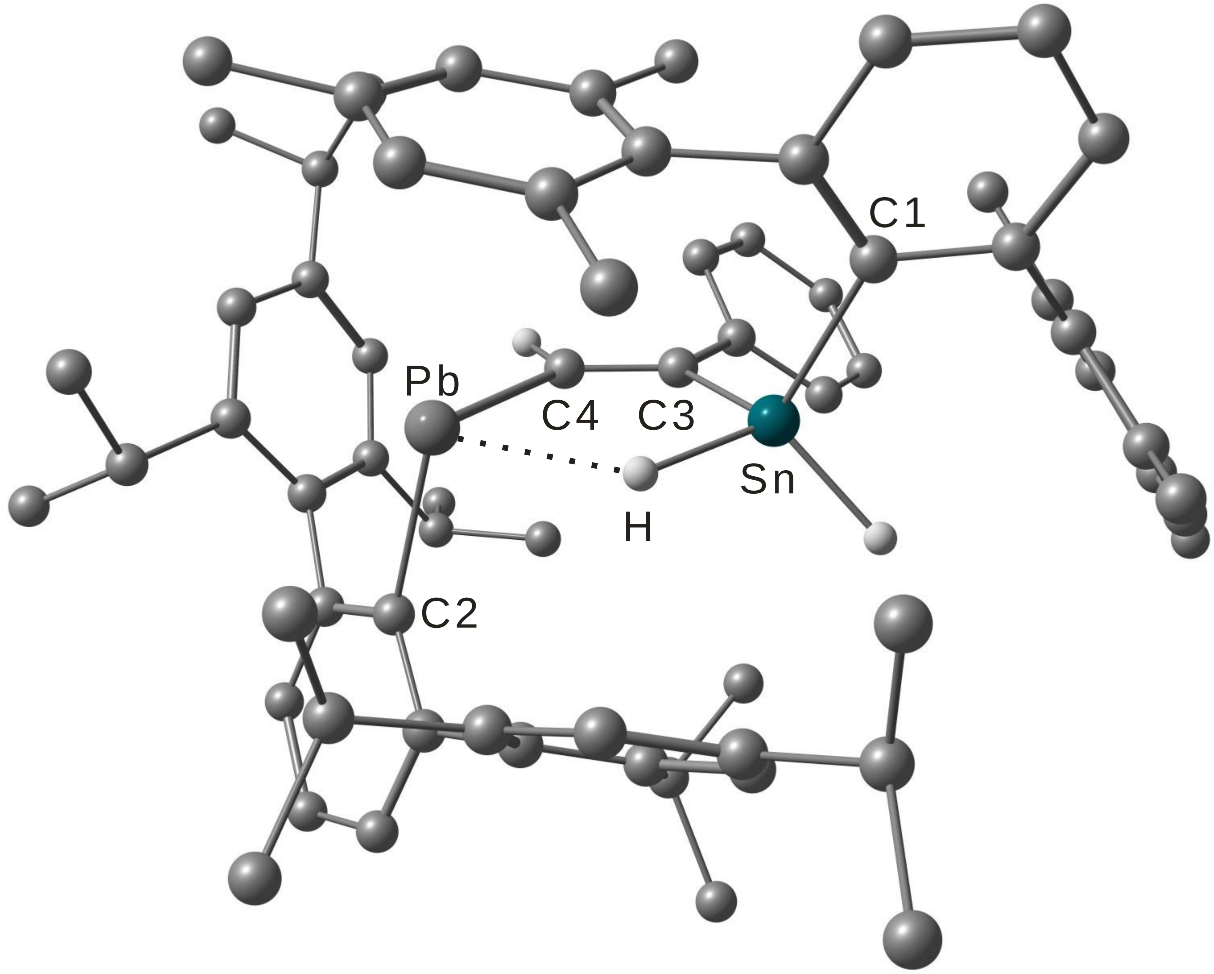

Figure S24. Optimized structure of Z-**8**. Hydrogen atoms except SnH_2 and vinyl CH were omitted for the sake of clarity.

Table S2. Selected distances (Å) and angles (°) in Z-**8**

**13**	Solid state structure	BP86 def2TZVP, ECPs Sn, Pb
C1–Sn	2.161(7)	2.175
C3–Sn	2.146(8)	2.170
C3–C4	1.336(9)	1.347
C2–Pb	2.318(7)	2.379
C4–Pb	2.274(7)	2.309
C1–Sn–C3	113.3(3)	115.0
C4–Pb–C2	99.1(3)	96.4
Sn–H		1.763
Pb–H		2.471

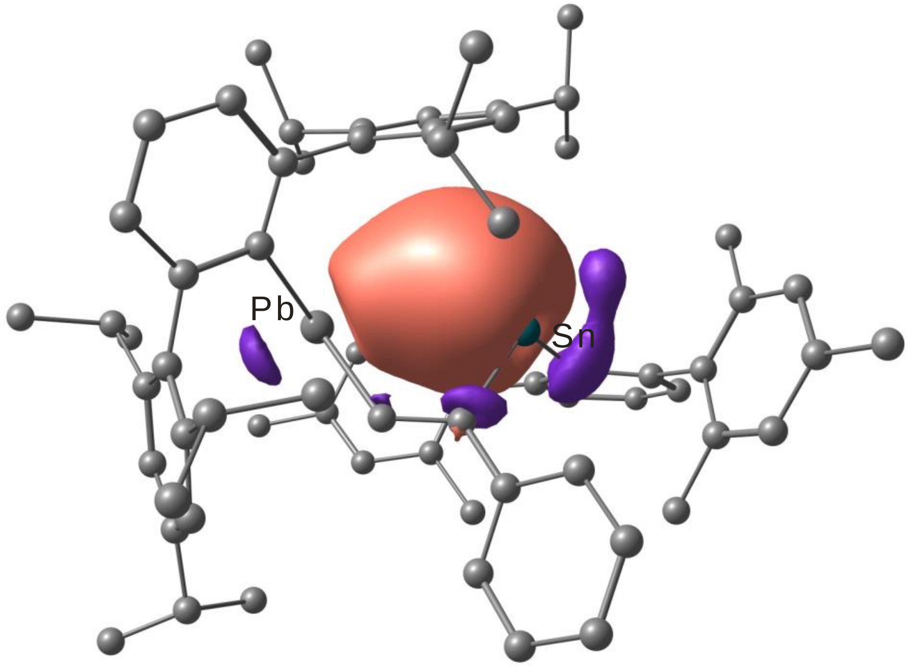

Figure S25. NLMO of Z-**8** showing the Sn-H-Pb interaction. Hydrogen atoms omitted for the sake of clarity. The Sn-H bond donates electron density into the empty p-orbital at Pb. NMLO: 63.23% H, 30.1 % Sn, 4.90% Pb (p-orbital)

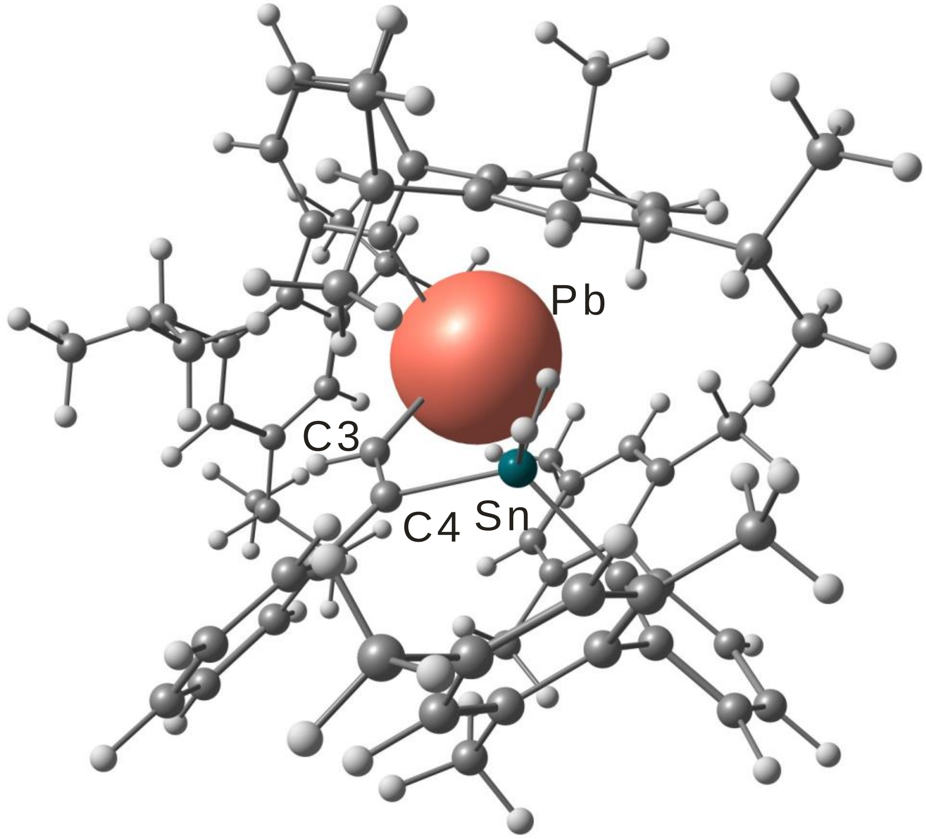

Figure S26. NLMO of Z-**8** showing the Pb-lone pair.

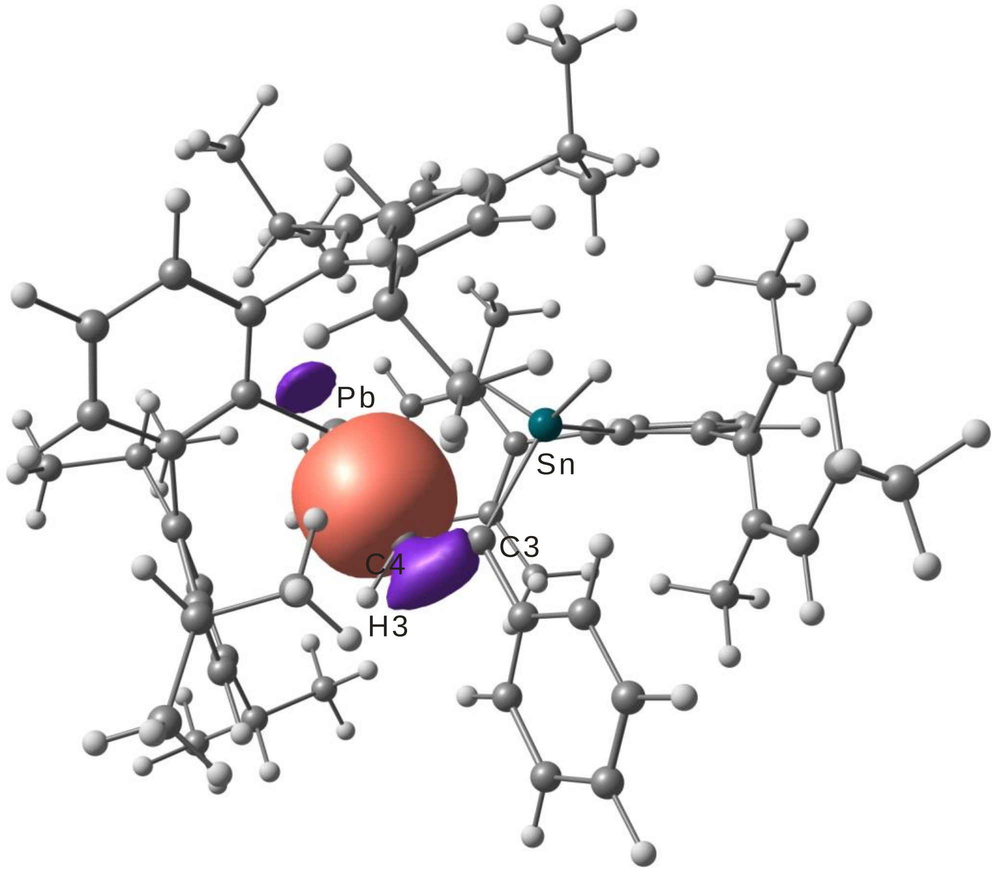

Figure S27. NLMO of Z-**8** showing the σ_p-Pb-C4 bond.

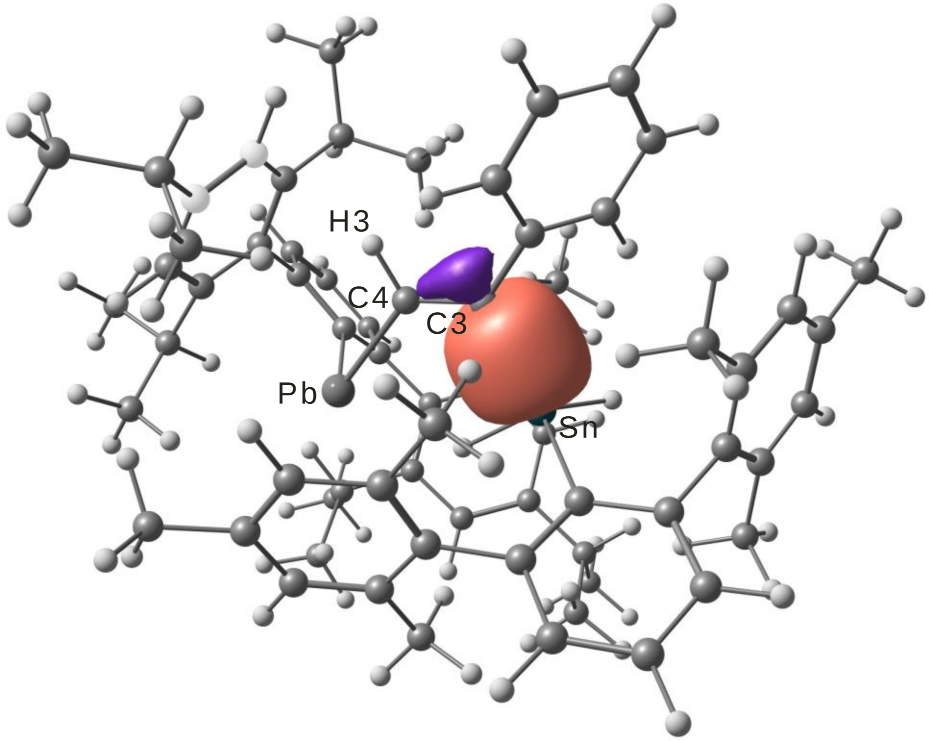

Figure S28. NLMO74 of Z-**8** showing the σ-Sn–C bond.

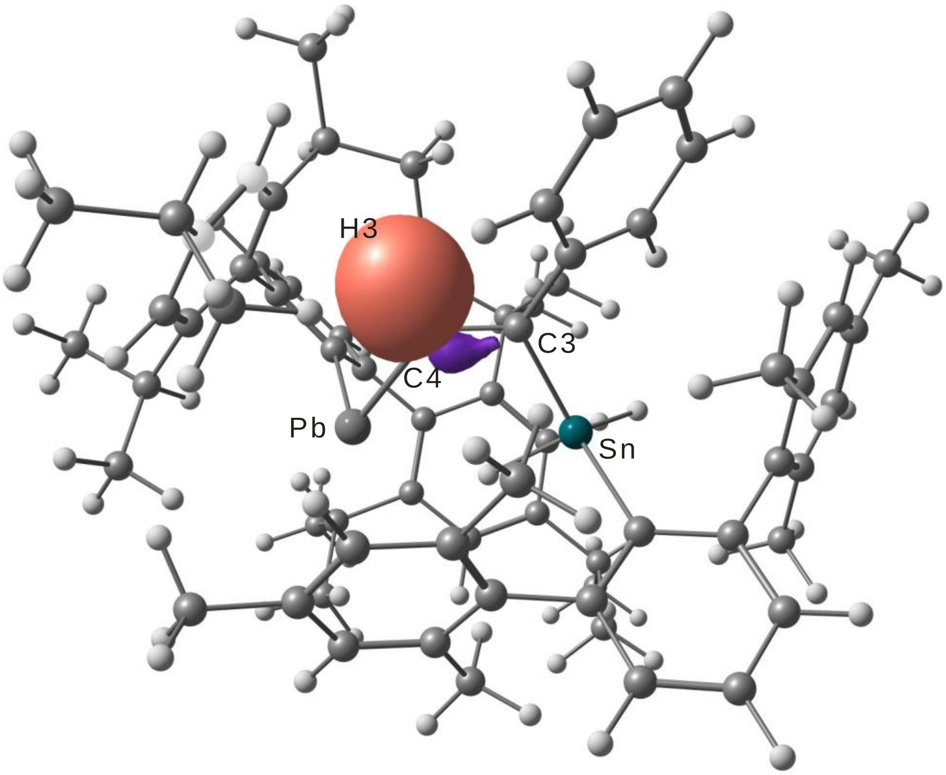

Figure S29. NLMO of Z-**8** showing the σ-C4–H3 bond.

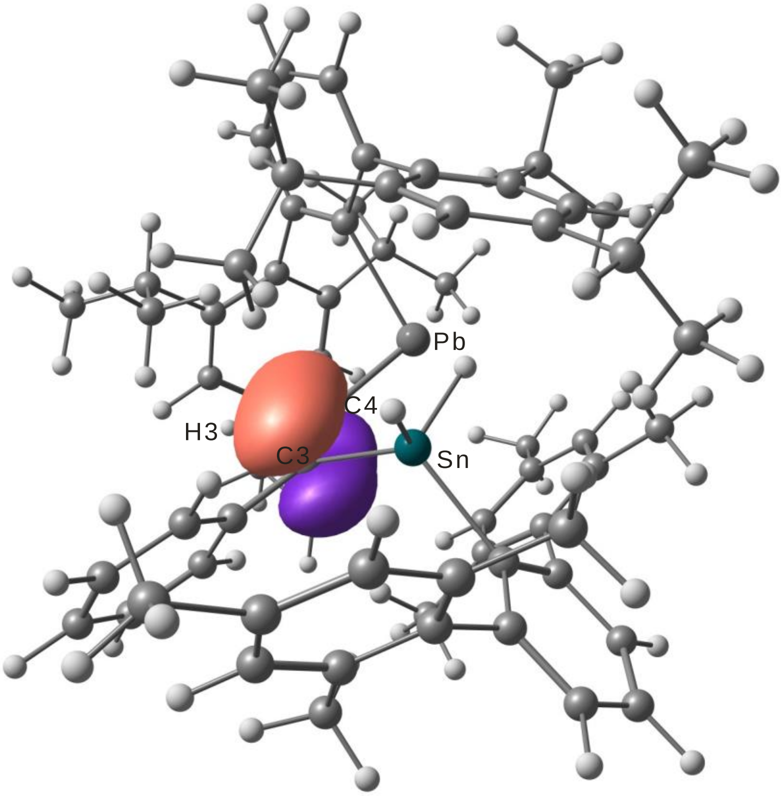

Figure S30. NLMO of Z-**8** showing the π_p-C3-C4 bond.

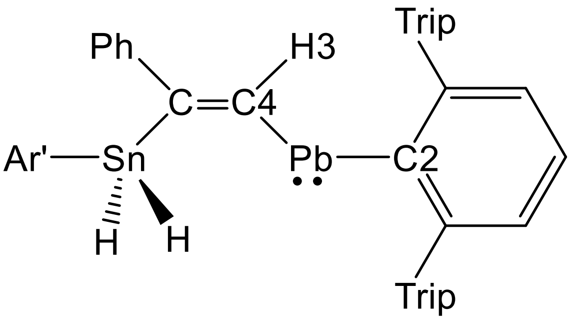

Table S3. Results of NMR chemical shift of compound Z-**8**.

	Exp.	ADF SO-ZORA	ReSpect 4c-mDKS
C2	259.6	258.0	250.1
C4	284.7	298.6	293.0
H3	11.50	11.0	10.1

Table S4. Contribution of various NMLOs to isotropic shieldings of Z-**8** (paramagnetic and SO parts)

NLMO	Pb lone pair	σ_p-Pb-C4	σ-Sn–C	σ-C4–H	π_p-C3–C4
C4		-223.9		-56.6	-56.3
H3	-1.2	-4.3	-1.4	-2.0	-1.8

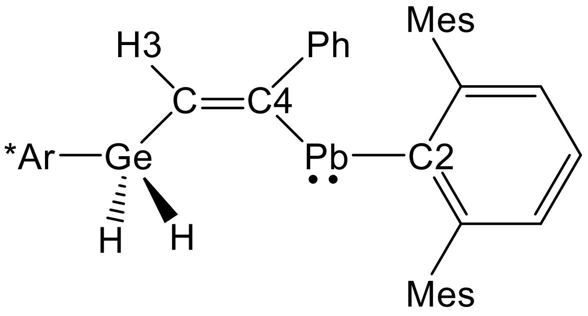

Table S5. Results of NMR chemical shift calculations for **5**.

	Exp.	ADF SO-ZORA	ADF Scalar-ZORA
C2	263.1	277.8	177.1
C4	273.6	308.7	210.2
H3	13.10	15.7	5.3

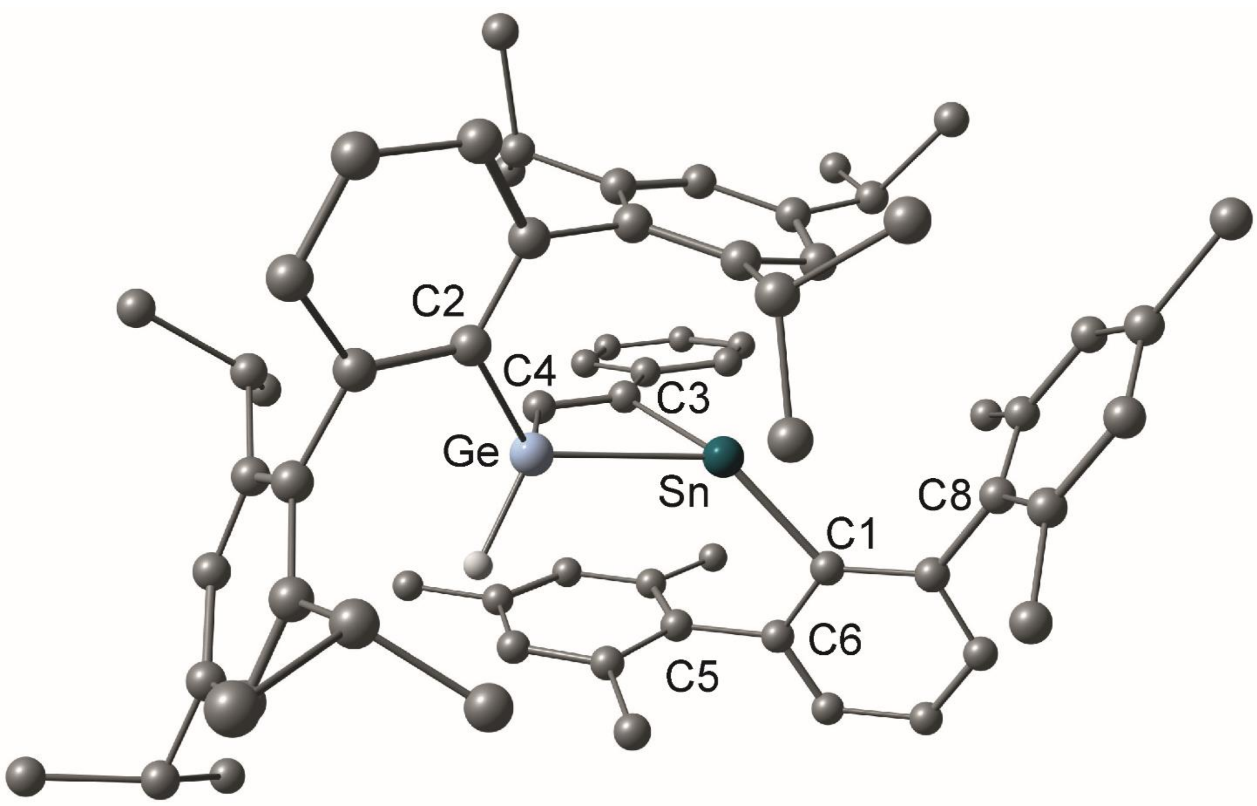

Figure S31. Optimized structure of cation of **9**.

Table S6. Selected distances (Å) and angles (°) in the cation of **9**

**14**	Solid state structure	BP86 def2TZVP, ECP Sn
Sn–Ge	2.669(1)	2.671
C1–Sn	2.145(2)	2.200
C3–Sn	2.138(2)	2.190
C3–C4	1.342(3)	1.356
C2–Ge	1.942(2)	1.966
C4–Ge	1.949(2)	1.977
C5–Sn	2.765(2)	3.036
C1–Sn–C3	123.3(1)	121.4
C1–Sn–Ge	157.9(1)	136.5
C3–C4–Ge	116.6(2)	114.8
C3–Sn–Ge	70.8(1)	70.3
C4–C3–Sn	100.9(2)	101.8
Sn–C1–C8	131.3(2)	127.7
Sn–C1–C6	106.5(2)	111.2

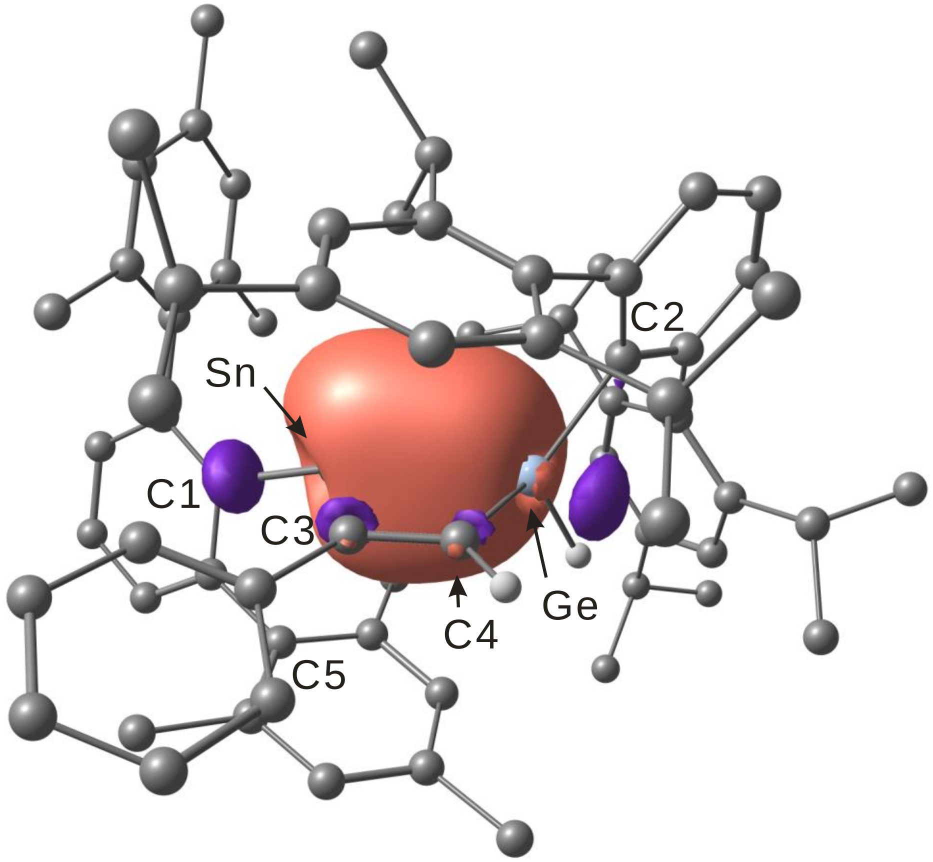

Figure S32. NLMO showing the σ-Sn-Ge bond of **9**.

NLMO: 43.6% Ge (24.7% s-orbital, 74.6% p-orbital), 52.2% Sn (62.5% s-orbital, 37.5% p-orbital)
NPA charges of Sn: 1.72, Ge: 1.05.

References

[1] a) L. J. Farrugia, *J. Appl. Crystallogr.* **1999**, *32*, 837-838; b) C. B. Hübschle, G. M. Sheldrick, B. Dittrich, *J. Appl. Crystallogr.* **2011**, *44*, 1281-1284; c) G. Sheldrick, *Acta Cryst., Sect. A* **2008**, *64*, 112-122; d) *Bruker AXS Inc. Madison, Wisconsin, USA, 2007* **2007**; e) G. Sheldrick, *SADABS, University of Göttingen, Germany, 2008*.

[2] R. K. Harris, E. D. Becker, S. M. C. d. Menezes, P. Granger, R. E. Hoffman, K. W. Zilm, *Pure Appl. Chem.* **2008**, *80*, 59-84.

[3] M. J. Frisch, G. W. Trucks, H. B. Schlegel, G. E. Scuseria, M. A. Robb, J. R. Cheeseman, G. Scalmani, V. Barone, B. Mennucci, G. A. Petersson, H. Nakatsuji, M. Caricato, X. Li, H. P. Hratchian, A. F. Izmaylov, J. Bloino, G. Zheng, J. L. Sonnenberg, M. Hada, M. Ehara, K. Toyota, R. Fukuda, J. Hasegawa, M. Ishida, T. Nakajima, Y. Honda, O. Kitao, H. Nakai, T. Vreven, J. J. A. Montgomery, J. E. Peralta, F. Ogliaro, M. Bearpark, J. J. Heyd, E. Brothers, K. N. Kudin, V. N. Staroverov, R. Kobayashi, J. Normand, K. Raghavachari, A. Rendell, J. C. Burant, S. S. Iyengar, J. Tomasi, M. Cossi, N. Rega, J. M. Millam, M. Klene, J. E. Knox, J. B. Cross, V. Bakken, C. Adamo, J. Jaramillo, R. Gomperts, R. E. Stratmann, O. Yazyev, A. J. Austin, R. Cammi, C. Pomelli, J. W. Ochterski, R. L. Martin, K. Morokuma, V. G. Zakrzewski, G. A. Voth, P. Salvador, J. J. D. S. Dapprich, A. D. Daniels,

Ö. Farkas, J. B. Foresman, J. V. Ortiz, J. Cioslowski, D. J. Fox, *Gaussian 09, Revision D.01, Gaussian, Inc., Wallingford CT* **2009**.

[4] a) J. P. Perdew, *Phys. Rev. B* **1986**, *33*, 8822-8824; b) A. D. Becke, *Phys. Rev. A* **1988**, *38*, 3098-3100.

[5] F. Weigend, R. Ahlrichs, *Phys. Chem. Chem. Phys.* **2005**, *7*, 3297-3305.

[6] a) F. Weigend, *Phys. Chem. Chem. Phys.* **2006**, *8*, 1057-1065; b) D. Andrae, U. Häussermann, M. Dolg, H. Stoll, H. Preuß, *Theoret. Chim. Acta* **1990**, *77*, 123-141; c) A. Bergner, M. Dolg, W. Küchle, H. Stoll, H. Preuß, *Mol. Phys.* **1993**, *80*, 1431-1441; d) B. Metz, H. Stoll, M. Dolg, *J. Chem. Phys.* **2000**, *113*, 2563-2569.

[7] a) S. Grimme, S. Ehrlich, L. Goerigk, *J. Comput. Chem.* **2011**, *32*, 1456-1465; b) S. Grimme, J. Antony, S. Ehrlich, H. Krieg, *J. Chem. Phys.* **2010**, *132*, 154104-154119.

[8] a) E. D. Glendening, C. R. Landis, F. Weinhold, *J. Comput. Chem.* **2013**, *34*, 1429-1437; b) E. D. Glendening, J. K. Badenhoop, A. E. Reed, J. E. Carpenter, J. A. Bohmann, C. M. Morales, C. R. Landis, F. Weinhold, *NBO 6, Theoretical Chemistry Institute, University of Wisconsin, Madison* **2013**; c) A. E. Reed, R. B. Weinstock, F. Weinhold, *J. Chem. Phys.* **1985**, *83*, 735-746.

[9] G. A. Zhurko, CHEMCRAFT (http://www.chemcraftprog.com).

[10] a) Vrije Universiteit, Amsterdam, The Netherlands, htpp://www.scm.com; b) S. K. Wolff, T. Ziegler, E. van Lenthe, E. J. Baerends, *J. Chem. Phys.* **1999**, *110*, 7689-7698; c) S. K. Wolff, T. Ziegler, *J. Chem. Phys.* **1998**, *109*, 895-905.

[11] J. Autschbach, *Mol. Phys.* **2013**, *111*, 2544-2554.

[12] J. Autschbach, *J. Chem. Phys.* **2008**, *128*, 164112.

[13] ReSpect 5.1.0 (2019), Relativistic spectroscopy DFT program of authors M. Repisky, S. Komorovsky, V. G. Malkin, O. L. Malkina, M. Kaupp, K. Ruud, with contributions from R. Bast, R. Di Remigio, U. Ekstrom, M. Kadek, S. Knecht, L. Konecny, E. Malkin, I. Malkin Ondik.

[14] W. Kutzelnigg, U. Fleischer, M. Schindler, Springer Berlin Heidelberg, Berlin, Heidelberg, **1991**, pp. 165-262.

[15] K. G. Dyall, *Theor. Chem. Account* **2006**, *115*, 441-447.
